# A stem acrodontan lizard in the Cretaceous of Brazil revises early lizard evolution in Gondwana

**DOI:** 10.1038/ncomms9149

**Published:** 2015-08-26

**Authors:** Tiago R. Simões, Everton Wilner, Michael W. Caldwell, Luiz C. Weinschütz, Alexander W. A. Kellner

**Affiliations:** 1 Department of Biological Sciences, University of Alberta, Edmonton, Alberta, Canada T6G2E9; 2 Centro Paleontológico da UnC (CENPALEO), Universidade do Contestado, Mafra, Santa Catarina, Brazil CEP 89300-000; 3 Department of Earth and Atmospheric Sciences, University of Alberta, Edmonton, Alberta, Canada T6G2E9; 4 Laboratory of Systematics and Taphonomy of Fossil Vertebrates, Departamento de Geologia e Paleontologia, Museu Nacional/Universidade Federal do Rio de Janeiro, Quinta da Boa Vista s/n, São Cristóvão, Rio de Janeiro, Brazil CEP 20940-040

## Abstract

Iguanians are one of the most diverse groups of extant lizards (>1,700 species) with acrodontan iguanians dominating in the Old World, and non-acrodontans in the New World. A new lizard species presented herein is the first acrodontan from South America, indicating acrodontans radiated throughout Gondwana much earlier than previously thought, and that some of the first South American lizards were more closely related to their counterparts in Africa and Asia than to the modern fauna of South America. This suggests both groups of iguanians achieved a worldwide distribution before the final breakup of Pangaea. At some point, non-acrodontans replaced acrodontans and became the only iguanians in the Americas, contrary to what happened on most of the Old World. This discovery also expands the diversity of Cretaceous lizards in South America, which with recent findings, suggests sphenodontians were not the dominant lepidosaurs in that continent as previously hypothesized.

## Abstract (web summary)

Iguanians are a diverse group of lizards. Here, the authors report an acrodontan iguanian from the Late Cretaceous of Brazil, which suggests that this group achieved a global distribution during the Mesozoic but was replaced by non-acrodontans in the Americas.

## Graphical abstract

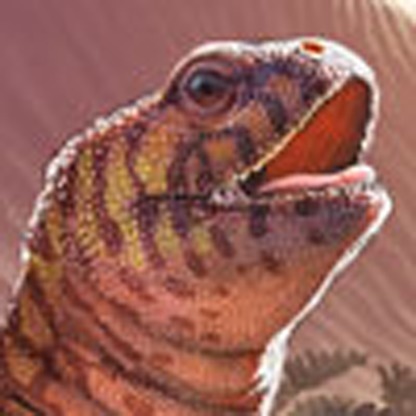

Squamates (lizards, snakes and amphisbaenians) are the most speciose extant group of reptiles, represented by more than 9,000 living species, and iguanians are one of the most diverse group of lizards globally, with more than 1,700 species^1^. Acrodontan iguanians are characterized by unique jaw features among lizards, as well as an evolutionary trend towards tooth placement at the apex of the jaws and fusion to it (acrodonty and pleuroacrodonty), whereas non-acrodontans (iguanids, tropidurids, among others) are pleurodont, with teeth attached to the lingual wall of the jaws. Among extant taxa, while acrodontans have an Old World distribution, non-acrodontan iguanians dominate the New World, as well as Madagascar and a few Pacific islands^2^. The origins of these two groups, with their almost disjunct distributions, and the dominance of non-acrodontans in the Americas have been the subject of great conjectures and debate^3^,^4^,^5^,^6^,^7^,^8^. The difficulty in providing answers to these questions is due to a poor fossil record worldwide during the time of origin of squamates (Early-Mid Mesozoic), and during the entire Mesozoic of Gondwana—nine valid species of lizards^9^, versus ∼150 species in Laurasia (data compiled from^7^ and several subsequent publications).

Here we report on the first known acrodontan iguanian lizard from South America, the New World component of ancient Gondwana, recovered from a new locality in Brazil dated as Late Cretaceous. This discovery overturns long held hypotheses of the evolution and palaeobiogeography of modern iguanian lizards and provides important insights into the early evolution of lizards in South America.

## Results

### Systematic palaeontology

       Squamata Oppel, 1811

       Acrodonta Cope, 1864

   *Gueragama sulamericana* gen. et sp. nov.

 **Etymology**. ‘Guera', meaning ‘ancient' (native Brazilian Tupi-Guarani); ‘agama'(gender feminine) in reference to agamid lizards; ‘sulamericana', meaning ‘from South America' (Portuguese).

**Figure 1:**
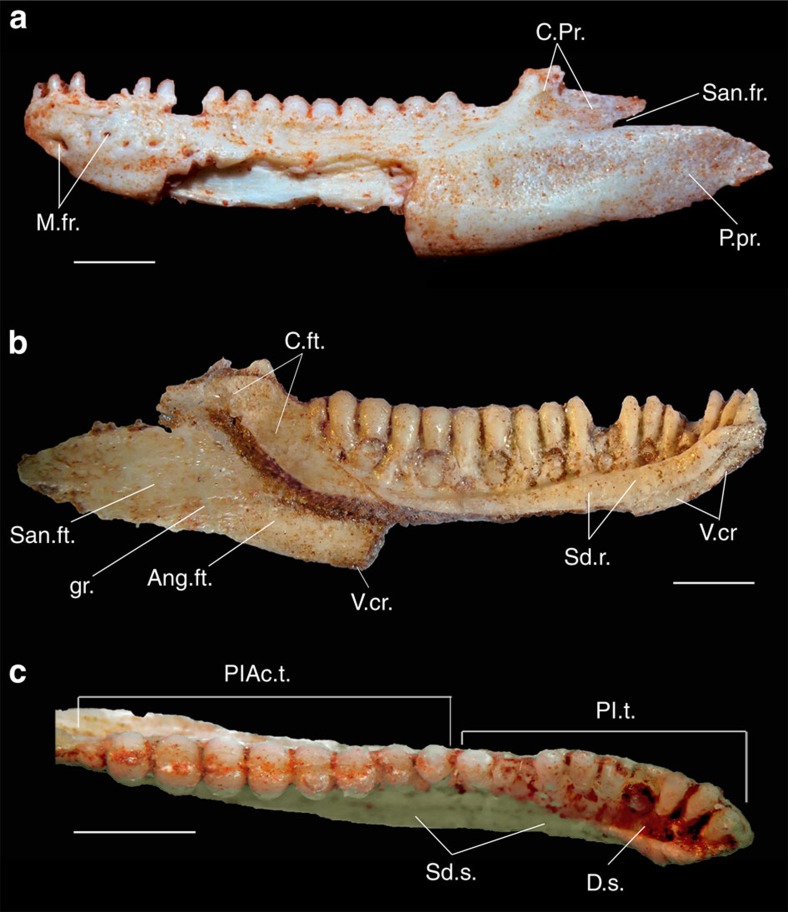
Holotype of *Gueragama sulamericana.* CP.V 2187 in (**a**) labial, (**b**) lingual and (**c**) occlusal views. Ang.ft., angular fac; C.ft., coronoid facet; C.Pr., coronoid process; D.s., dental sulcus; gr., groove; M.fr., mental foramina; P.pr., posterior process; Pl.t., pleurodont teeth; PlAc.t., pleuroacrodont teeth; San.fr., surangular foramen; San.ft. surangular facet; Sd.r., subdental ridge; Sd.s., subdental shelf; V.cr., ventral crest of dentary. Scale bars, 2 mm.

 **Holotype**. CP.V 2187, partial lower jaw (Fig. 1), CENPALEO—Universidade do Contestado, Santa Catarina, Brazil.

 **Additional material**. CP.V 2188 (unprepared fragments of possible maxillary and teeth).

 **Type locality and horizon**. Cruzeiro do Oeste, Paraná State, Brazil; Goio-Erê Formation, Caiuá Group, Bauru Basin; Turonian-Campanian, Late Cretaceous^10^.

 **Diagnosis**. Stem acrodontan species separated from all other squamates by the following combination of characters: coronoid process of dentary with dorsal and posteriorly elongate component; posterior process of dentary undivided and extending well beyond level of coronoid process; presence of subdental shelf; dental sulcus present anteriorly; no splenial articulatory facet on dentary (splenial either small or absent); large facet for anteromedial process of coronoid on dentary; angular extending anterior to posteriormost tooth; anterior teeth pleurodont, peg-like, with pointed and laterally compressed apices; posterior marginal teeth: pleuroacrodont, straight, posteriorly increasing in size (except for the last tooth) and labiolingually expanded, increasing degree of ankylosis with subdental shelf and labial wall of dentary posteriorly and with apical wear creating labial and lingual shearing crests; replacement pits lingual to functional tooth.

**Description**. The preserved dentary has a convex ventral border that is partially broken in its midsection, and bears six mental foramina laterally. The coronoid process would have covered the coronoid eminence laterally, probably reaching to or beyond its posterior margin. In medial view, the anterior tip of the dentary has a horizontally elongate symphysial flat surface that would have butted against its right counterpart, and is barely indented ventrally by the Meckelian canal. The subdental shelf has a medial ridge that diminishes in height posteriorly and which delimits a dental sulcus anteriorly that is not filled with cementum (as opposed to teiids) and is mostly empty. Despite the ventral border of the dentary being broken in its midsection, the ventral crest of the dentary is preserved and visible in medial view ventral to the subdental shelf, anteriorly and posteriorly. It does not extend medially, and does not contact the subdental ridge, even in its deepest part on the anteriormost section of the dentary. This indicates that the Meckelian canal was fully open medially, even in the region where the subdental shelf was deepest. Posterior to the last tooth position there is a large facet for the coronoid anteriomedial process, and posterior and laterally to this, facets for the surangular and angular bones (Fig. 1). Inside the Meckelian canal, the intramandibular septum is not seen posteriorly. There is an excavation on the dorsolateral surface of the dentary, which creates a posterodorsal crest that extends onto the coronoid process, as observed in the extant agamid *Uromastyx.* There is no facet for the splenial medially on the subdental shelf.

**Figure 2:**
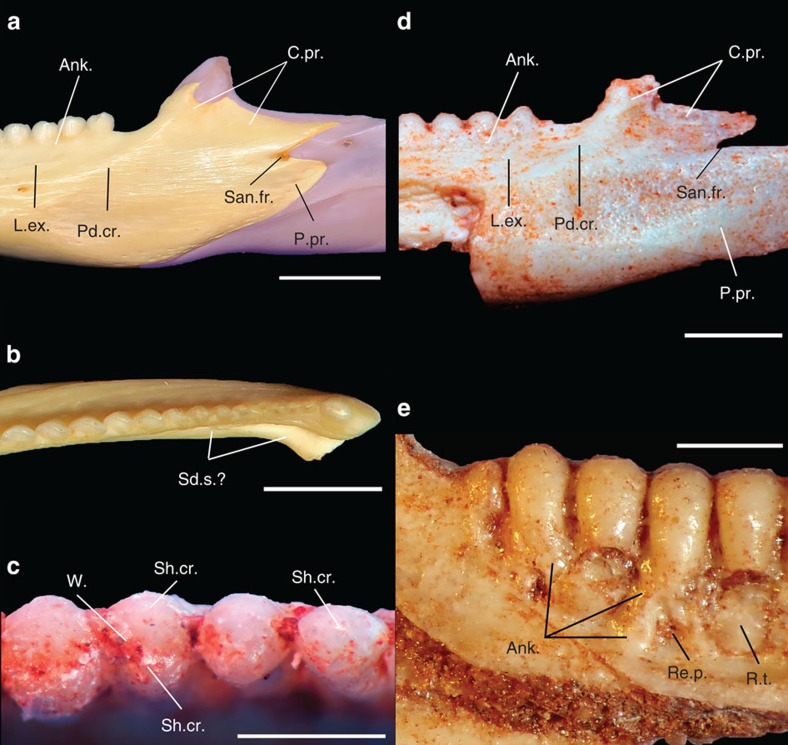
Details of *Gueragama sulamericana* and comparisons. Details of the lower jaw of *Uromastyx acanthinurus* (extant acrodontan—MCZ 27382) in labial (**a**) and occlusal (**b**) views. Scale bars, 5 mm. Details on the dentition and dentary of *Gueragama sulamericana* (CP.V 2,187) in occlusal (**c**), labial (**d**) and lingual (**e**) views. Scale bars, 1 mm. Ank., tooth ankylosis to dentary bone; L.ex., labial excavation on dentary; Pd.cr., posterodorsally ascending crest; Re.p., resorption pits; Re.t., replacement tooth; Sd.s., subdental shelf; Sh.cr., shearing crest; W., mediodistal wear facet.

There are 18 tooth positions, with most teeth preserved *in situ* and displaying moderate heterodonty. The anteriormost five teeth display no obvious ankylosis to the labial wall. However, there is an increasing degree of ankylosis to the labial wall of the dentary and the subdental shelf after the eighth tooth, with the dorsal crest of the dentary eventually becoming indistinguishable from the teeth (Fig. 2d). Posterior teeth are also positioned more dorsally on the jaw relative to anterior teeth, with the labial wall of the dentary (and its dorsal crest) not being visible in dorsal view. Thus, the anteriormost teeth can be classified as pleurodont, and the posterior series as pleuroacrodont. The resorption pits are elliptical, having a wider diameter in the apicobasal axis.

The teeth in the middle and posterior sections of the jaw are straight and closely spaced, whereas the three anteriormost teeth are slightly inclined anteriorly. Resorption pits are observed throughout the dentary, indicating replacement was active. The anterior teeth also have pointed apices that are laterally compressed, forming relatively sharp anterior and posterior ridges. The teeth posterior to the eighth tooth position become gradually different, bearing a sagittaly oriented wear facet at their apices, leaving a labial and a lingual shearing crest and forming a molariform series.

### Systematic comparisons to other lepidosaurs

Some features of the jaw of *Gueragama*, such as a posteriorly elongated coronoid process of the dentary, and an undivided and elongate posterior process, are similar to those found in most rhynchocephalians. However, rhynchocephalians usually have a fully acrodont dentition and show no tooth replacement, making them very distinct from *Gueragama*. The early rhynchocephalians *Gephyrosaurus*^11^ and *Diphyodontosaurus*^12^ from the Late Triassic and Early Jurassic of Britain, nevertheless, show pleurodont tooth attachment and pleurodont+acrodont attachment, respectively. Yet, these and all other rhynchocephalians differ from *Gueragama* and crown acrodontans by having a closed Meckelian fossa, or nearly closed in *Gephyrosaurus*. Even in the latter condition, the ventral crest of the dentary is curved medially and is also deep anteriorly, as in other rhynchocephalians, unlike *Gueragama* and crown acrodontans. *Gephyrosaurus* and *Diphyodontosaurus* also show the primitive lepidosaurian condition of an elongate jaw with a high tooth count^11^,^12^ (reaching up to 40 dentary teeth in *Gephyrosaurus*^13^), whereas the jaw in *Gueragama* and crown acrodontans is much shorter with a lower tooth count. The anterior inferior alveolar foramen is usually located at the level of the coronoid process in rhynchocephalians (or slightly anteriorly in some cases), but it is more anterior in acrodontans^14^. *Gueragama* does not have the anterior inferior alveolar foramen at the level of the coronoid and it is inferred to be located well anteriorly (its probable location is covered by matrix and is too fragile for preparation). Finally, *Gephyrosaurus* and *Diphyodontosaurus* have an oval symphysis split by the Meckelian canal, and later rhynchocephalians have a vertically elongate symphysis. *Gueragama*, as in acrodontans (except chamaeleonids), has the symphysis elongated and nearly horizontal, differing from the conditions observed in rhynchocephalians. All these factors indicate *Gueragama* is not a rhynchocephalian, and is instead, an acrodontan.

Within Squamata, the lack of plicidentine infolding coupled with straight teeth, and the long and undivided posterior process of the dentary, makes *Gueragama* different from all members of the Anguimorpha. The medially open Meckelian canal also indicates *Gueragama* is neither a gekkotan nor a xantusiid, which possess a medially closed Meckelian canal via fusion of the dentary subdental shelf to the ventral border of the dentary. An open Meckelian canal is a common feature amongst most lizards classically classified in the ‘Scincomorpha'. However, among scincomorphs, *Gueragama* differs from teiioids by having a long undivided posterior process of the dentary, a well-developed and dorsally oriented component of the coronoid process and by having an elliptical (wider diameter in the apicobasal axis) rather than semi-circular resorption pits and lacking deep deposits of cementum. From lacertids, it differs by its long posterior and coronoid processes of the dentary. Xantusiids, cordyloids, scincids (including the fossil contogenoids from the Late Cretaceous of North America), as well as some other fossil taxa possibly closely related to them, such as *Globaura* and *Carusia* from the Late Cretaceous of East Asia, do have an elongate posterior process of the dentary that is undivided. However, in these groups the posterior process usually differs from the condition observed in *Gueragama* by being located on the ventral margin of the jaw, at some distance from the coronoid process and the anterior surangular foramen, being separated from them by the surangular. In some forms in these families, this process can be straight (for example, the scincid *Tiliqua* and contogenoids, as well as the xantusiid *Palaeoxantusia* from the Eocene of Wyoming and North Dakota). Yet, and most importantly, all these groups differ from *Gueragama*, by possessing a Meckelian canal that is closed anteriorly (with subdental shelf and ventral crests in contact medially), or totally closed, as in xantusiids; a coronoid process of the dentary that extends mostly dorsally, exposing the coronoid labial process posteriorly to it; anterior and posterior ridges on the lingual side of marginal teeth (for contogenoids); an elongate splenial (apart from xantusiids); and having tall chisel-like teeth, usually with crown striations (mostly in scincids). *Gueragama* also differs from the Late Cretaceous borioteiioids by having a posteriorly elongate coronoid process (much shorter in borioteiioids), the elongate posterior process of the dentary (very reduced in borioteiioids) and a reduced/absent splenial (elongate in borioteiioids).

*Gueragama sulamericana* shares with iguanian lizards a subdental shelf, closely spaced teeth, and the replacement teeth positioned lingually. Whereas some families of iguanians have a closed Meckelian canal, the new species possesses an open Meckelian canal, which is also the case for acrodontans, and some species of *Oplurus*, and *Phrynosoma*, for instance. The new species shares with acrodontan lizards numerous other features (see Table 1) such as an undivided and straight posterior process of the dentary, separated from the coronoid process by a small gap through which the anterior surangular foramen opened; a coronoid process with an elongate posterior component, which covers the lateral surface of the coronoid bone; a dorsolateral excavation on the labial margin of the dentary, producing a posterodorsal crest connecting it to the coronoid process; splenial either small or absent; an anteriorly elongate angular facet on the dentary, indicating that element extended anteriorly to the posteriormost teeth. When compared with all fossil and extant squamates, the combination of these features is observed only in acrodontan lizards (Figs 1 and 2). The new taxon is most similar to the basal acrodontan group Leiolepidinae, especially *Uromastyx*, including the morphology of the coronoid process that is subdivided into a dorsal and a posterior elongate component in *Uromastyx* (Fig. 2; Table 1).

### Comparative anatomy of acrodontans and priscagamids

Crown acrodontans and the Priscagamidae, from the Late Cretaceous of Mongolia^15^, form a monophyletic group according to most authors^15^,^16^,^17^,^18^,^19^. The dentition of *G. sulamericana* is more similar to primitive priscagamids by having its posterior teeth predominantly placed on the medial side of the jaw. For instance, *Pleurodontagama*^15^,^20^, *Flaviagama* and *Mimeosaurus tugrikinesis*^16^ have an exclusively pleurodont dentition, as does *Ctenomastax*^21^, a putative sister taxon to priscagamids+acrodontans^18^. *Priscagama* had both pleurodont and acrodont teeth posteriorly. Among acrodontans, transitional dentitions that are still not fully acrodont occur in Eocene species from Mongolia, such as *Khaichinsaurus* and *Lentisaurus*^22^, as well as a few modern forms (for example, *Uromastyx* and *Calotes*^23^). The tooth attachment modes in these taxa suggest acrodonty could have evolved as a derived condition within Iguania, and that the priscagamids (and some acrodontans) represent transitional forms evolving towards that dental condition^20^. In the priscagamids *Pleurodontagama* and *Morunasius*, some resorption pits indicating tooth replacement are also retained^20^.

*G. sulamericana* displays an increasing degree of dental ankylosis of the posterior teeth, the lack of it in the anteriormost teeth, and the presence of a dental sulcus as in *Priscagama* and *Pleurodontagama*^15^,^20^. Tooth replacement is still present, as in *Pleurodontagama* and *Morunasius.* Furthermore, the variation in tooth placement on the jaw of *G. sulamericana* is also seen in priscagamids and acrodontans. *G. sulamericana* also displays close packing of the teeth, which resembles the primitive iguanian condition, as observed in *Ctenomastax*. However, *G. sulamericana* preserves 18 teeth, differing from the higher number of teeth in non-acrodontan iguanian dentaries (20–35), and matching the tooth count of acrodontans and some priscagamids, which varies between 15 and 20 (ref. 24). Thus, *Gueragama* displays unique lower jaw features of acrodontans as well as their tooth count, but still bears dental characteristics of other iguanians that are also retained in priscagamids.

### Phylogeny

**Figure 3:**
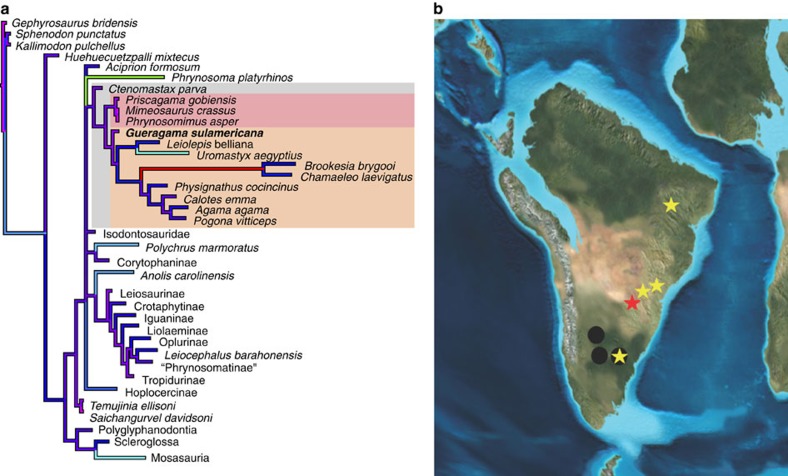
Phylogenetic position of *Gueragama sulamericana* among other squamates, and lepidosaur distribution in the Cretaceous of South America. (**a**) Strict consensus tree of 373 most parsimonious trees of 5,287 steps each (consistency index=0.2012; retention index=0.7714). Branches are proportional to lengths, and emphasized by a colour gradient of increasing branch length as follows: pink, purple, blue, cyan, green, yellow and red. The following clades are denoted: Priscagamidae (pink box), Acrodonta (light orange box), Priscagamidae+Acrodonta+*Ctenomastax* (grey box). The extremely long branch leading to chamaeleons (*Brookesia* and *Chamaeleo*) suggests either the absence of basal fossil forms, or rapid evolutionary rates. (**b**) Between the Aptian/Albian (112 million years ago (mya) and the Campanian (83 mya), sphenodontians were present in northern Patagonia, in the provinces of Chubut (Tres Cerros), Río Negro (Los Alamitos, Cerro Tortuga, Cerro Bonaparte and La Buitrera) and Neuquén (El Chocón), represented by black circles. Lizards were present in the state of Ceará in northeastern Brazil (Araripe Basin), as well as in the southeastern/southern states of Minas Gerais (Peirópolis), São Paulo (Marília and Presidente Prudente) and Paraná (Cruzeiro do Oeste), and in the province of Río Negro (Cinco Saltos and La Buitrera), Argentina, represented by stars. The red star indicates the type locality of *G. sulamericana*.

We inferred the phylogenetic position of *G. sulamericana* in a data set representative of all major squamate groups^18^ (see Methods and Supplementary Data 1), and obtained a well resolved strict consensus tree topology and an unambiguous placement for the new taxon (Fig. 3a). *Gueragama* was found as a stem acrodontan, being more closely related to crown acrodontans than to the Priscagamidae and *Ctenomastax*. *Gueragama* breaks the long branch between priscagamids and acrodontans found a previous analysis^18^, providing clues for character evolution along the stem lineage of Acrodonta.

Despite the limited number of characters that could be scored for *Gueragama* in this character matrix, taxon incompleteness should not be an *a priori* criterion for not including a taxon in a phylogenetic analysis. Retrieving an unambiguous positioning for a taxon with few scorable characters, and good resolution for the entire tree, depends on the taxon possessing the key synapomorphies that are necessary for its correct placement^25^,^26^, and this cannot be predicted before the analysis^27^. The inclusion of *Gueragama* in the matrix of Gauthier *et al.*^18^ supports this hypothesis. *Gueragama* was found within the clade formed by *Ctenomastax*, along with priscagamids and acrodonts, within the acrodontan clade, but outside of the crown, supporting the transitional position for this taxon.

In a preliminary analysis without the inclusion of *Gueragama*, the branch between priscagamids and extant acrodontans was 18 steps long. The inclusion of *Gueragama* broke this relatively long branch leading to the acrodontan crown clade into two shorter branches: one of three steps (subtending *Gueragama* and crown acrodontans) and another of three steps (subtending the crown clade)—Supplementary Note 1. The inclusion of this fossil form does not change the relationship between major clades of squamates obtained before its inclusion, but helps to understand the sequence of character evolution leading to the evolution of the peculiar jaw and teeth features that characterize acrodontans amongst all other squamates. Furthermore, breaking long branches makes the overall analysis more accurate, as biases due to long-branch attraction are diminished. The long branch obtained by Gauthier *et al.*^18^ for the chamaeleonids (*Chamaeleo* and *Brookesia*) was also inferred from the present analysis. This configuration is expected given the high degree of morphological specialization of the members of this clade in relation to its sister taxa, and the absence of intermediate fossil forms representing the transition to that morphotype. It is likely, given the ancestry of the clade Acrodonta (back into the Jurassic^14^), the lack of intermediate fossils leading to crown chamaeleonids and the paucity of African and Malagasy fossils, that this long branch will be broken by the discovery of stem chamaeleonids, as has occurred for acrodontans after the inclusion of *Gueragama*. Whether stem chamaeleonids first diversified in Madagascar, Africa or other regions may not affect current ideas on the origin of the modern fauna. Crown chamaeleonids (that have been estimated to have originated in Madagascar^4^) may be the last survivors of a previously more diverse chamaeleonid total clade, the origin of which is still unknown.

### Palaeohabitat

**Figure 4:**
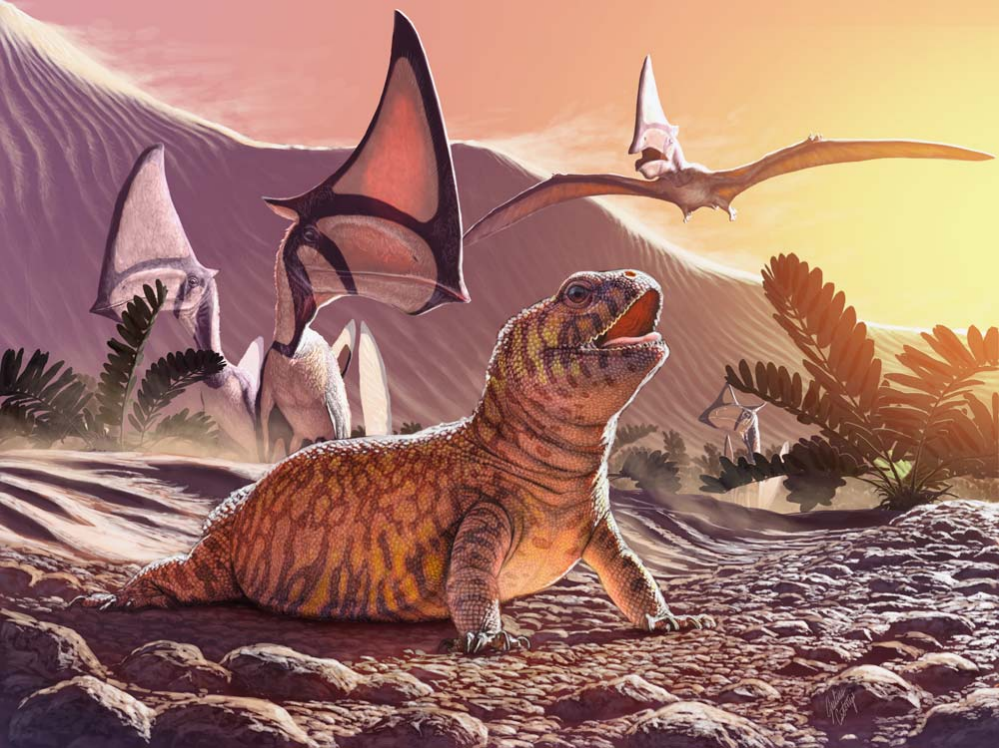
Life reconstruction of *Gueragama sulamericana* in its palaeohabitat. As the extant agamid *Uromastyx* in Africa, *G. sulamericana* also inhabited a desert environment. The new species was found in an ancient oasis along with pterosaurs of the species *Caiuajara dobruskii.* Reconstruction created by J. Csotonyi.

*G. sulamericana* lived in an arid to desert environment, the Caiuá desert belonging to the Bauru Group in Southeastern/Southern Brazil during the Late Cretaceous. Ichnofossils of large dinosaurs are known from the central areas of that desert, indicating that large animals were able to survive there. This was probably due to seasonal water availability and the interdune wetland characteristics of the Goio-Erê Formation^28^. The Goio-Erê oasis probably supported some plant life, though plant fossils are still unknown. Pterosaurs were also abundant in that region, represented by hundreds of bones of the tapejarid *Caiuajara dobruskii* that have been found in the same locality as *Gueragama*^10^ (Fig. 4). Like modern agamid lizards living in arid regions, *Gueragama* probably lived in burrows to avoid extreme temperatures during at least part of the day.

## Discussion

*G. sulamericana* provides fundamental insights on the acquisition of the peculiar lower jaw and tooth morphologies of modern acrodontans. Tooth ankylosis in the early branching priscagamids with pleurodont tooth attachment, and in *G. sulamericana* with pleurodont and pleuroacrodont attachment, indicates that ankylosis evolved before the dorsal placement of the posterior teeth that is characteristic of the dentition of modern agamids and chamaeleonids. In fact, *Uromastyx* and some other agamids also have a dorsomedial, rather than a strictly dorsal (for example, chamaeleonids), placement of the posterior teeth on the lower jaw, despite full ankylosis of the posterior adult teeth. The position of *Priscagama* as an early branching priscagamid in previous works^18^,^19^ also suggests that fully acrodont attachment evolved independently within the priscagamids and acrodontans, now further supported by *G. sulamericana*. Furthermore, *Uromastyx*, a later branching agamid, possesses an anteromedial projection of the dentary, which seems to be the remnant of a subdental shelf (Fig. 2), still present in *G. sulamericana.* The strong apical wear facets that create a sagittal groove in *G. sulamericana* are unusual for extant acrodontans, which usually possess this feature on the labial surface of the dentary^29^. However, a similar wear pattern has recently been identified in an Oligocene specimen that represents the oldest known acrodontan from Africa, and which also shares many features with *Uromastyx*^30^.

The oldest known acrodontans are from the Early-Middle Jurassic of India^14^. Furthermore, morphological phylogenetic data suggests Iguania (including acrodontans) and Scleroglossa separated before the breakup of Pangea^7^, which would explain the worldwide distribution reported for non-acrodontan iguanians by the Late Cretaceous^7^,^21^,^31^. *Gueragama* indicates that a worldwide distribution was also achieved by acrodontan lizards by the Late Cretaceous, occurring not only in East Gondwana and East Laurasia, but also reaching West Gondwana during the Mesozoic. This reinforces the idea of an early radiation and wide distribution for all iguanians, and also implies that the acrodontan presence in Africa could be much older than the current oldest record^30^. Finally, *Gueragama* indicates that acrodontan dispersal through Gondwanan continents first occurred before the final breakup of Gondwana, and not by later dispersal events, thus contradicting previous hypotheses^6^,^8^. Therefore, it becomes clear that iguanians underwent a worldwide radiation in the Mesozoic. Whether the modern distribution of these faunas is a result of this early radiation, with subsequent extinction in some areas (for example, acrodontans in South America), or the result of subsequent Cenozoic dispersal, remains to be established. However, our findings, along with previous ones^4^,^5^, suggest both factors were important.

It is clear that at some point in time, a diverse non-acrodontan iguanian fauna eventually replaced *Gueragama* and other potential acrodontans in South America. These two large groups of iguanians may well have come into contact during the Late Cretaceous in South America^32^,^33^, similar to what occurred in East Asia for non-acrodontans and priscagamids during this same time period^15^,^21^. Acrodontan and non-acrodontan iguanians share very similar ecologies^2^ and their current almost exclusively non-overlapping distributions worldwide suggest competitive exclusion as a possible explanation for their current distributions. Alternatively, the Late Cretaceous extinction event could have paved the way for non-acrodontan iguanian dominance in South America.

*Gueragama* also suggests a new scenario for early acrodontan radiation, never considered before: that the stem acrodontan lineage could have evolved in West Gondwana, rather than in East Gondwana. Whether acrodontans originated in, or radiated into South America during the Mesozoic, is still unclear. Further sampling from both South America and other Gondwanan localities are necessary to appropriately address this question. Yet, *Gueragama* does indicate that at least some of the oldest known lizards in South America include species that are more closely related to the extant fauna of Old World continents, rather than to the modern fauna of South America.

In spite of limited knowledge of the squamate fossil record in South America, new finds are continuing to expand our understanding of squamate evolution in South America, especially in Brazil^9^,^32^,^34^ and Argentina^35^,^36^. The diversity of lizards in the Cretaceous of South America is higher than previously thought, and is not dominated by sphenodontians as was previously suggested for that time period^37^. Rather, the previous lack of lizard records seems to have been a collection bias from southern latitudes in South America. The current pattern of distribution suggests that different major lizard groups were already present in Northeastern and Southern Brazil (this work and^9^,^32^,^33^), where no sphenodontians are currently known (Fig. 3b), while sphenodontians were still abundant, but apparently restricted to more southern latitudes (Southernmost Brazil and Argentina).

## Methods

### Anatomical nomenclature

Nomenclature throughout the text follows two main sources^38^,^39^.

### Phylogenetic analysis

To phylogenetically infer the systematic position of *G. sulamericana* among squamates, we included it into the data matrix of Gauthier *et al.*^18^ with the taxon scoring corrections performed by Simões *et al.*^9^. The search parameters involved the New Technology algorithms implemented in T.N.T.^40^, as these are the more appropriate ones for retrieving trees from the largest number of possible local optima for data matrices larger than 100 taxa. The algorithms used were Sectorial Search, Ratchet and Tree Fusing, followed by a traditional heuristic search using the protocol performed by Simões *et al.*^9^. This method allowed us to obtain shorter most parsimonious trees and in larger numbers than the original run by Gauthier *et al.*^18^. During this process, additional incorrect character-state scores were identified for some taxa, and corrections were made:

Ch. 367—*Zonosaurus*, which was used by Gauthier *et al.*^18^ as an example for character-state 367 (2), as well as other cordylids and xantusiids, was mistakenly scored with state 367 (1) and thus rescored herein as 367 (2). Despite illustrating *Trogonophis* with character-state 367 (3), this and most other amphisbaenians were scored as either ‘0' or ‘1'. Because characters-states ‘1' and ‘2' differ in the topography of the coronoid process, and ‘2' and ‘3' in its degree of development, we scored amphisbaenians according to the topography of that process (either ‘1' or ‘2'). Character-state ‘3' should be treated under a separate character if one desires to code for degree of development, and rescored for all taxa, but this extra character seems to have intermediate states that prevent its scoring without creating arbitrary and biased categories. Thus, we did not attempt to create this extra character.

Primitively, in *Sphenodon* and other rhynchocephalians, the dentary bears a posterodorsal coronoid process and a more ventrally placed posterior process^41^ with the former lying above the level of the mandibular foramen and the latter beneath it. Such a division between a posterodorsal coronoid process and a posterior process is also present in the basalmost rhynchocephalians *Gephyrosaurus*^11^ and in the early squamate *Huehuecuetzpalli.* That posterior process in squamates is also located ventral to a mandibular foramen, the anterior surangular foramen. Within squamates, it is common for the posterior process to become divided into a dorsal surangular process, and a more ventral angular process, with both usually lying ventral to the anterior surangular foramen^41^,^42^. Thus, the topography of the posterior process(es) relative to the mandibular/surangular foramen is generally conservative, and it is an important topography marker to establish the primary homology of these processes based on similarity. Acrodontans have a coronoid process covering the base of the coronoid bone, above the anterior surangular foramen, and a single undivided posterior process ventral to that foramen, similar to rhynchocephalians and to other lepidosaurians, as previously recognized by Borsuk-Bialynicka^42^ and Evans *et al.*^14^. However, Gauthier *et al.*^18^ scored acrodontans with state 367 (0), as if they lacked a coronoid process. Thus, we rescored their state from ‘0' to ‘2'.

Character 367 was also treated as ordered in the character description, but in the character assumption in the data matrix it was treated as unordered by Gauthier *et al.*^18^. We maintained this character as unordered. All the forementioned changes are summarized as follows: 1→2: *Tepexisaurus tepexii*, *Cricosaura typica*, *Lepidophyma flavimaculatum*, *Palaeoxantusia sp.*, *Xantusia vigilis*, *Platysaurus imperator*, *Cordylus mossambicus*, *Zonosaurus ornatus*, *Cordylosaurus subtesselatus*, *Myrmecodaptria microphagosa*, *Carusia intermedia*, *Globaura venusta*, *Eoxanta lacertifrons*, *Bipes canaliculatus; Bipes biporus; Geocalamus acutus; Amphisbaena fuliginosa*, 0→2: *Sphenodon punctatus*, *Kallimodon pulchellus*, *Gephyrosaurus bridensis*, *Huehuecuetzpalli mixtecus*, *Leiolepis belliana*, *Uromastyx aegyptius*, *Brookesia brygooi*, *Chamaeleo laevigatus*, *Physignathus cocincinus*, *Agama agama*, *Calotes emma*, *Pogona vitticeps*, *Adamisaurus magnidentatus*, *Polyglyphanodon sternbergi*; 0→?: *Gobinatus arenosus*; 1→?: *Hymenosaurus clarki*; 0→1: *Spathorhynchus fossorium*, *Dyticonastis rensbergeri*, *Rhineura floridana.*

Ch. 369—The posterior termination of the dentary in some acrodontan taxa is clearly posterior to the level of the coronoid apex, as illustrated for *Leiocephalus* for character-state 369(2). Thus, we rescored these taxa: 1→2: *Uromastyx aegyptius*, *Brookesia brygooi*, *Chamaeleo laevigatus*, *Physignathus cocincinus*, *Agama agama*.

Ch. 372—*Gephyrosaurus* and *Sphenodon* were scored as having an open Meckelian canal medially [372(0)], as seen among acrodontans. However, *Gephyrosaurus* was described^11^ as having the ventral margin of the canal contacting the dorsal tooth-bearing portion, closing the canal, but not fusing. Thus, this taxon needs to be scored with character-state 372(2). The same is observed in *Sphenodon.*

### Nomenclatural acts

This published work and the nomenclatural acts it contains have been registered in ZooBank, the proposed online registration system for the International Code of Zoological Nomenclature. The ZooBank LSIDs (Life Science Identifiers) can be resolved and the associated information viewed through any standard web browser by appending the Life Science Identifier to the prefix ‘ http://zoobank.org/'. The LSIDs for this publication are: urn:lsid:zoobank.org:act:428CA59D-1BA3-4A29-914D-5D0D29FE4CCD; urn:lsid:zoobank.org:act:1D135173-7E42-47E1-8A70-4ABEA9D677FD.

## Additional information

**How to cite this article:** Simões, T. R. *et al.* A stem acrodontan lizard in the Cretaceous of Brazil revises early lizard evolution in Gondwana. *Nat. Commun.* 6:8149 doi: 10.1038/ncomms9149 (2015).

## Supplementary Material

Supplementary Information Supplementary Note 1

Supplementary Data 1 Updated morphological data matrix

## Figures and Tables

**Table 1:** Distribution of diagnostic features present in *Gueragama sulamericana* sp. nov. and other acrodontans+priscagamids.

**Anatomical traits**	**Priscagamids**	***G. sulamericana***	Leiolepidinae*	**Other acrodontans**
1. Fully open Meckelian canal	Present	Present	Present	Present
2. Elongate angular facet on dentary (angular anterior to posteriormost tooth)	Present	Present	Present	Present
3. Heterodonty (disconsidering the extreme wear that erodes the anterior teeth in some acrodontans during ontogeny)	Present	Present	Present	Present
4. Posterior teeth ankylosis to dentary lingual wall	Present	Present	Present	Present
5. Posterior teeth positioned apicolingually on the jaw	Present	Present	Present	Variably present
6. Number of teeth ranging between 15–20 on the lower jaw	Variably present	Present	Present	Present
7. Close packing of teeth	Variably present	Present	Variably present	Variably present
8. Undivided and straight posterior process of the dentary	Absent	Present	Present	Present
9. Posterior process separated by a small gap from coronoid process (aperture for surangular foramen)	Absent	Present	Present	Present
10. Coronoid process with an elongate posterior component	Absent	Present	Present	Present
11. Dorsolateral excavation on the labial margin of the lower jaw, producing posterodorsally ascending crest on coronoid process anterior margin	Absent	Present	Present	Variably present
12. No articulatory facet for the splenial on the medial margin of the dentary (splenial small or absent)	Absent	Present	Present	Present

*G. sulamericana*, *Gueragama sulamericana.*

^*^Leiolepidinae (*Uromastyx* and *Leiolepis*) is taken separately from other acrodontans, due to its key position, generally taken as an early branching acrodontan. Leiolepidines have important features peculiar to them amongst extant forms, which are variably present in other acrodontans, and are also retained in *G. sulamericana* and other fossil acrodontans.
