# Supplementary material for: A stem acrodontan lizard in the Cretaceous of Brazil revises early lizard evolution in Gondwana
Source: Nat Commun. 2015 Aug 26;6:8149. doi: 10.1038/ncomms9149 (PMC4560825; doi:10.1038/ncomms9149)
Supplement: Supplementary Information — Supplementary Note 1 [file ncomms9149-s1.pdf]

Supplementary Note 1

Synapomorphies for the Priscagamidae, Acrodonta and closely related taxa:

Priscagamidae+Acrodonta+*Ctenomastax*:

Char. 62: 0 --> 2
Char. 372: 23 --> 0
Char. 413: 1 --> 0
Char. 417: 0 --> 1
Char. 419: 3 --> 2

Priscagamidae+Acrodonta:

Char. 3: 0 --> 1
Char. 25: 0 --> 1
Char. 120: 0 --> 1
Char. 136: 0 --> 1
Char. 285: 1 --> 0
Char. 364: 0 --> 1
Char. 415: 0 --> 1

Priscagamidae:

Char. 94: 2 --> 0
Char. 146: 0 --> 1
Char. 156: 0 --> 1
Char. 375: 1 --> 3
Char. 572: 12 --> 3

Acrodonta:

Char. 364: 1 --> 2
Char. 367: 0 --> 2
Char. 369: 01 --> 2

Acrodonta (- *Gueragama*):

Char. 366: 0 --> 2
Char. 423: 0 --> 1
Char. 430: 0 --> 1

Uromastyx + *Leiolepis*:

Char. 82: 0 --> 2
Char. 147: 0 --> 1
Char. 204: 0 --> 1
Char. 275: 0 --> 2
Char. 420: 3 --> 2
Char. 424: 0 --> 1
Char. 439: 0 --> 1
Char. 483: 1 --> 0

Char. 484: 0 --> 1
Char. 498: 1 --> 0
Char. 572: 1 --> 0

Chamaeleonidae:

Char. 20: 0 --> 1
Char. 50: 0 --> 1
Char. 56: 1 --> 2
Char. 57: 1 --> 3
Char. 98: 0 --> 1
Char. 99: 0 --> 1
Char. 114: 1 --> 0
Char. 116: 2 --> 0
Char. 129: 0 --> 2
Char. 130: 1 --> 2
Char. 134: 0 --> 1
Char. 173: 0 --> 1
Char. 177: 1 --> 2
Char. 180: 0 --> 1
Char. 182: 2 --> 3
Char. 183: 0 --> 1
Char. 188: 0 --> 1
Char. 194: 0 --> 2
Char. 196: 0 --> 1
Char. 212: 0 --> 1
Char. 290: 0 --> 1
Char. 301: 0 --> 2
Char. 311: 0 --> 1
Char. 328: 0 --> 2
Char. 338: 0 --> 1
Char. 350: 0 --> 1
Char. 360: 1 --> 0
Char. 361: 4 --> 3
Char. 374: 0 --> 1
Char. 383: 0 --> 1
Char. 404: 0 --> 1
Char. 417: 1 --> 0
Char. 439: 0 --> 2
Char. 445: 1 --> 3
Char. 446: 0 --> 1
Char. 454: 01 --> 2

Char. 459: 0 --> 1
Char. 463: 3 --> 2
Char. 477: 1 --> 0
Char. 481: 1 --> 0
Char. 483: 1 --> 2
Char. 487: 0 --> 2
Char. 488: 0 --> 1
Char. 493: 1 --> 0
Char. 495: 1 --> 0
Char. 499: 0 --> 1
Char. 505: 0 --> 1
Char. 513: 1 --> 0
Char. 514: 0 --> 1
Char. 517: 0 --> 1
Char. 518: 0 --> 2
Char. 521: 0 --> 1
Char. 522: 0 --> 1
Char. 523: 0 --> 1
Char. 530: 0 --> 1
Char. 531: 0 --> 1
Char. 532: 0 --> 1
Char. 536: 0 --> 1
Char. 539: 1 --> 0
Char. 540: 0 --> 1
Char. 545: 0 --> 1
Char. 546: 1 --> 0
Char. 547: 0 --> 1
Char. 549: 0 --> 1
Char. 558: 0 --> 1
Char. 563: 0 --> 1
Char. 568: 0 --> 1
Char. 569: 0 --> 1
