# Supplementary material for: A stem acrodontan lizard in the Cretaceous of Brazil revises early lizard evolution in Gondwana
Source: Nat Commun. 2015 Aug 26;6:8149. doi: 10.1038/ncomms9149 (PMC4560825; doi:10.1038/ncomms9149)
Supplement: Supplementary Data 1 — Updated morphological data matrix [file ncomms9149-s2.docx]

**Supplementary Data 1**

**Updated data matrix of Gauthier *et al.* with the inclusion of *Gueragama* and the character scoring corrections detailed in the Methods section:**

nstates 8 ;

xread 'Data saved from TNT'

610 193

Sphenodon_punctatus 00000?021000000?00000000000000000??00000000?0?0??0000000000??0012000000?0?000000000?1?0000??1?00000?00000000000?0100000200100000000000001?000000?00000000100000000?001??????????0?010000100000001000000?00000??00??010000??000?000000100000?0??100?0000000?0?202000?000??01??0?00?0000?0000000?0?0000000000000000010?0?00??0???00?0000?0??000000??000000?0??0?100000000040?2?0202002?1?????00100?010000?00?010010?01??????0?0?100022201000???1??000000010??01000000000100000002?0?01010???0?10000??????000000?000?000000000?000000000??010?0000010?0000000?0?0000?0000????0000000?00?0000??0000?0000?000?01000000000000000000?0000

Kallimodon_pulchellus 00000??0100000??00000??0000?00000??000000?????????0?0000000??0002000000?0????0?0????1??0?0??1?0?000?0000000??00???00??0?00?00???00?0?????????000??0?000???00?00?00??01??????????0?0?0???????????????????????????????????????00?0?????1???0??????00?0?00000?0?20200???0?0??1??0?????00????????????????????00000???????????????????????0?1??????????0???0???????????00000????2?02020???1?????00?0??00?00??00????01??0100??0?000???00022010000??1??00000????????????????0000000001?0?01000??????0?????????10??00?001?0?00??0????0?00??00??01000??00?0?????????0?00000?00?????00000?1?00?000???0000?0000?0????????????????????????????

Gephyrosaurus_bridensis 00000?001000000000000?000000?00000010000000?0?0??0000000000??000100000000?000000000?0?0100??0?0?000001000000?00001010001?00000000000000000000000?00000001010000?00?001??????????0?00000??0100??????0??0????0???????000000?0?00?0?0000000?00?0?0100?0000000?0?001000?00000000000?00000100?0?000?0?0????00?00000??????????????????0??000??????01?00000100????????00000000140?100202002?1?????0000?0000000?00??00001?00000000000014004440000000?000?0000???????????????????????000?0?010000000????????????00??00000??000000000?000001000??00000??0000?????????????????000????0?0???1??0???????1??????????????????????????????????????

Huehuecuetzpalli_mixtecus 0?0?012?01??00?00000??0?000010000??1000000??0??100000?00100??0200000002?0?00000000?00?0100??000?0?0?0100000???0???01?0???00?000?00?000??00??000100??20000?2??00?00?00000000?00001?10120??0?00?1??????????????????????1????????????????????????????????????????????????0??????0???????????????????0????1?100000?????????????????00?????????????????????0?????????0??0000?40?0?020000??0?????01????01?020?10???000?0????????000??00?433000??0?0????0000???????1????????0000000002?0?01000???0?1000??10??0000001?10??0000??00???00010?00??00000??001???00?0???0?00001000?????0110001?00?000???1000?0000?0????????????????????????????

Ctenomastax_parva 10000?000000000?0000000?000010000??10000000?0?0100000001120??2110000000?0?0001?0000?0?0100??0?0?000?01101000?01??202000000000000010000000000000100002000002?000000000000000?00001?101?0??0?00?????????????????????0?????????0?????0??1?00??????010?000?001?0?01??????00???1??0000?0000?0?00010?0?0??0?1???00?0????????????????????0000???????????00???0???????????00000130?000000000001??0??1?01001?02??10?000?00000000000100?001023200000000000??010?????????????????????????????1????????????????????????????????????????????????????????????????????????????????????????????????????????1000?0000??????????????????????????????

Priscagama_gobiensis 10100?000000000?00000?01100011000??10000000?0?0300000001120??2??0?000?2?0?0001?0000???0100??0000000?011010000010?20200010000000?0100000100000001010020000021000000000000000?000010101200?0?00????0?000100?000??0??00?0000??000?000?0?1?0000????010?0000001?0?01?1?1?0000?010?0000?0000?0?00000?0?0100012??0010??0?0000000??????00?0000????00010??00??00???0??0??0100000140?1000100000030000010010011020010?000000000000000100?1010232000000??000020?0???????????????????????0???0?1????????????????????????????????????????????????????????????????????????????????????????????????????????3000?0000??????????????????????????????

Mimeosaurus_crassus 10100?000?0??00???0????????0???00??10000000?0?020000000112???2??0?000?2?0?000??0000???0100??0?0?000?01101000?010?202?0010000000?0100000100000001010020100021000?00000000000?000010101?0??0?00?0????0?010???0??????0?????????0?????????000???????10?000?0?????????????000?0???0?00?0000?0?0000??0?0100?12??00000???0??0?0???????00?000??????001????????0?????????01?00??1???1000?0000?03000???00??????2??1????01??00???????100?1010222?0??0???000?20?0?????????????????????????????1????????????????????????????????????????????????????????????????????????????????????????????????????????3000?0000??????????????????????????????

Phrynosomimus_asper 1?10????0000000?00000001?000??000??10000?0????02000000011????21?0?000?2?0?000??0000???0100??000?0?0?01101000???0??0??0??0000000?01?000???????001?1?02010002?000?00??0???0????????????????0??????????0??????????0??0????????????????????????????????????0??????1???????????1??0000?0000?0?00000?0???????2?????????????0??????????0?0000?????00???????????????????0??00???????0?????00??3?00??????????????1?????????0???????1???????????00000??000??????????????????????????????????1?????????????????????????????????????00?????????????????????????????????????????????????????????????????3000?0000??????????????????????????????

Leiolepis_belliana 102000300000000?00000011100010000??10000000?0?0300000001120??32000000?2?0?000110020???0100??0000000?011010000020120200010000000?0100000100000000001120000220000000000000000?000010101100102000000000001000010??01?0100000??000?000000100000?0??010?0000001?0?01?111?0000?11??0000?2000?0?00000?0?01001121000000001000000000100?00?0000?0??00010100000?00010100010100000140?20220100000000??010010010010110?000101000000000100?1010223011000??1???201001101001001100000000000003?0?10030???0?10001101??0000001010100200110010[13]00[01]11000[01]100000000010001120111000000100000010011001010000000??0000?0000?00200100100000000000?00100010

Uromastyx_aegyptius 102000[23]00200?00?00110??1100010000??1?000000?0?0400000001120??1?????00?2?0?000?10020???0100??0200000?011010000020120?00000010000?010000011?000001?01010001020000000000000000?000010101200?02200000000001000010??01?0010000??000?000000101000?0??210?0000003?0?01?111?0000?11??0000?2000?0?00010?0?00000121000000001000000000100?00?0000?0??00010100010001010110010100000040?20220200000000??010012010010110?00000?000000000000?00?0122011000??1???001001101001000000000000000003?0?10030???0?100011010?0000001011100000110010000110000[01]000000[01]10010001120111000000100000012011001[01]10000000??0000?0000?0020010????????????????100??0

Brookesia_brygooi 10200??200?0000?10010??1100000000??10000000?0?0??1000002300???????000???0?000400100????100??0200011?00?0100?0?20100000020000000?2203?1011?100000?00010001120000?0000000???0?10?02001131000240000?2?1???????????????10000????00?00?000110000?0??110?0000001?0?01?111?000??11??0000?0000?0?001?0?0?1?????2000020000010?01100?100?00?0000?2??00010101000?00010101010100000030?202202000?1??0??0?0112010010?10?000001001??????110?1000132010000??1???201002200?031??011002000010002?0?10030?????00000?210?2110100?00111?????1????000010012?01110000011111121110101001010111012011101011001011??3000?0000?00?0012????????????????100??0

Chamaeleo_laevigatus 102000320??0000?00010??1100010000??10000000?0?0??10000023?0???????000???0?000400100????100??12?0011?2??1?00?002?100000020000000?2200?1011?100000?10020000020000?0000000???0?10?020011310?0?100000201???????????????10000????00?00?000?10000?0??110?0000001?0?01?111?000??11??0000?0000?0?00100?0?1?????2?0002000010000100??110?00?0000?2??0001010100110?010101010100000030?2?2202000?1?????01010?010010?10??0000?001??????01??1000032010000??1???20100220[01]?031??0????2000030002?0?10030?????00000?300[34]2110100?00111?????1????000010012?011101?0021111121??01020010101?1???0111110?1002011??1000?0000?0030010????????????????10???0

Physignathus_cocincinus 102000320000000?00000?11100000000??10000000?0?0300000001120??32000000?2?0?001?001?0???0100??0?00000?011010100020110200020000000?0100000100100000000010000210000000000000000?000010101200002000001000001000000??01?0000000??000?000000120000?0??110?0000001?0?01?111?0000?11??0000?0000?0?00100?0?01000121000000101000000000100?00?0000?0??00010100011001010110010100000140?2022?200000000??010012011000110?00000?000001000111?1010122010000??1???201000200?01011000000100000003?0?10030???0?1000101002001000111011020001001010001000001000000000100011201110000001000010?0011001010000000??0000?0000?002001001?00000?00?0?00100010

Agama_agama 1020004200?0000?01000?11100010000??10000000?0?0300000001120??32000000?2?0?000?00100???0100??0100000?011010100020110200030000000?0100000100100000?00010001210000000000000000?000010101200102010000000001000000??01?0000000??000?000000100000?0??010?0010001?0?01?111?0000?11??0000?0000?0?00100?0?0101?121000000101000000000100?00?0000?0??00010100000001010100010100000130?20220200000?0???010010010000110?0000000000010001?0?1010032010000??1???001000100?00011000001000000103?0?10030???0?100011100200101011100100000000100000100001100000000010001120111000000100001010011001000000000??1000?0000?002001001000000?00?0?0010???0

Calotes_emma 102000420000000?01000?11100010000??10000000?0?0300000001120??32000000?2?0?000400100???0100??0100000?01101010002?120200030000000?0100000?1?100000?00020001220000000000000000?000010101200002000001000001000000??01?0200000??000?000000120000?0??010?0010001?0?01?101?0000?11??0000?0000?0?00100?0?0101?121000000102000000000100?00?0000?0??00010101010001010100010100000140?20220100000000??010012010000110?00010100000100011101010132010000??1???001000100?01011000001000000103?0?10030?????10001120030010101110110000010010[12]00011000010000000001000112011100000010000[01]010011001000000000??1000?0000?0020010??????0????????0100??0

Pogona_vitticeps 102000420000000?00000?11100010000??10000000?0?0300000001120??32000000?2?0?000400100???0100??0000000?011010100020110200030000000?010000011?100000?00010001220000000000000000?000010101200102010000000001000000??01?0000000??000?000000100000?0??010?0010001?0?01?111?0000?11??0000?0000?0?00100?0?000101210000001020000000001?0?00?0000?0??00010100000001010100010100000140?20220100000000??010012010000110?00010000000100011101010132010000??1???001000?00?010000000?0100000103?0?10030???0?10001100?200?0001110110000000010100?101000?00000??0010001120111000000100000??0011001000000000??1000?0000?0020010010000000000000010???0

Temujinia_ellisoni ????????0?0????????????????0???00??1?000000?0?0100000001120??0110000000?0?0001?0000?0?0100??010?000?0110100000??????00000000000001?0?0000000000100002000002?0001000010000?0?00001?10120??0???????0????????????????0????????????????????0???????0?0?0000001?0?01?1?1??000?01??0000?0000?0?000?0?0?0?00????000100???000000??????0?0?0000?????00101?00?0?00??0?000?01??0001???0000000010020010010010011020010?00000?0000000001???0000?3200000000000?2000???????????????????????0???0?12???????????????????????????????????????????????????????????????????????????????????????????????????????1?00?0000??????????????????????????????

Saichangurvel_davidsoni ?0000???0000000?0000000??0001?00???100000??????2000???011????0110000000?0?00?1?0??0?0?01?0??000?000?011010000011?102?0000000000?01?00?0?0000000100??2000002?0001000010000?0?00001??01??0????????????????????????????????????????????????????????1???0????????????????0???????0?00?0000?0?????????0??00?2?00000???????????????????????????????????????????????????????00???????0?????00?0???0?0???01?0?0?1????0?0?000??0?0??01000003??000000??????20?????????1????????0000000?01???11020??????0?????????010??1?11??0???1??????0111??0???00000??001000???0??10??0?01?00?0????110????00???0???1000?0?00?0????????????????????????????

Isodontosaurus_gracilis 10000???00??000?0000000??00010000??10000000?0?0200000001121??1????000???1?000??0000?0?01?0??000?000?01101000?0?1?202000000000000110000?00000000100002000022?000?00??01??????????1?10120??0???????????????0????????0????????????????????0???????0????1???0?????1?1????0?0?01??0000?0000?0?000?0?0?0?00?12??0000?????00000????????0?0000?????0010??00???0??????0??0100000140?0020000010020000010012011020010?00010?0000000001???0000?2200000000000?2000???????????????????????0???0?1????????????????????????????????????????????????????????????????????????????????????????????????????????0000?0000??????????????????????????????

Zapsosaurus_sceliphros ????????????????0????????0?0???00??1?000000???0100000001121??1????000?2?0?000??0000????1?0??010?000?01101000?0????02??000?00000?01?000??00???0???????0?00?????0???00?00?000?00001?10??0??0????????????????????????0?????????0???????????????????????????????????1????0?0??1??0000?0000?0?00010?0?0??0?1???0000??????00??????????0?000??????001???00?0?00??0?????0?000??040?0000?00?3001?0????001001?02??1??0?01000000000001?????00??30000000?000?2000???????????????????????0???0?1????????????????????????????????????????????????????????????????????????????????????????????????????????0??0?0000??????????????????????????????

Polrussia_mongoliensis ???????????????????????????0???0???10000000???00000000011?1??1?????00???1?0001???00???01?0??020?000?01101000?0????02??000000000?01?000???0???001?0002000002?000?00000???????????1?10??0??0????????????????????????0????????????????????????????0?0??1???0?????101????000?000?0000?0000?0?????0?0?0??0?12??000???????00????????????0????????0010???????0????????????00001???0000?0103000000?01002001?020?10??0010?000?100001???0000?220000000000000000???????????????????????0???0?1????????????????????????????????????????????????????????????????????????????????????????????????????????0?00?0?00??????????????????????????????

Basiliscus_basiliscus 100000200000000?00000001000010000??10000000?0?0300000001120??2??0?000?2?0?000110000???0100??22?0000?0110200000111202000000000000010000000000000101002000002?00000000000?000?00001010120000?000000000001000000??01?0000000??000?000000110000?0??010?0000001?0?01?101?0000?00000000?0000?0000010?0?0100012100000000200000000?100?00?0000?0??00010100000001010000010100000140?0?00000030010?00010010012000010?0000000000000001010000033300000000000020000010??01001000000000000012?0?130?0?????100010[01]003010010111011021011000?10001000011000000[01]0010?0112011?00000010000?0?0011001[01]?0000000??1000?0000?0010010?????????0??????10?010

Corytophanes_cristatus 100000200020000?0000000??00011000??10000000?0?0200000001120??2??0?000?2?0?000110000???0100??22?0000?00102000001?1202000000000000010000000000000??1002000122?00000000000?000?000010101200102000000?00001000000??01?0?00000??000?000000110000?0??010?0000001?0?01?121?0000?01??0000?0000?000001??0?0001?12100000000110000000?100?00?0000????00010100000001010000010100000140?0000?0003001?000010?10012010010?00000?0000000001010000033300000000000?200000100?01011000000000000013?0?13030???1010000?[01]00[23]001000111001021001000?2000100000100000000010001120111000000100001010011001000000000??2000?0000?001001001000000?00?0?0010?010

Polychrus_marmoratus 100001200?20000?0000000?000011000??10000000?0?0?10000001120??2??0?000?2?0?000110000???0100??0200000?0111?00000101202000000000000010000000200000100012000012?000000000000000?0000101012001021000000000010000?0???1?0000000??000?000000100000?0??010?0000003?0?01?121?0000?00000000?0000?0?00010?0?0101?12100000000110?000000100?00?0000?0??0001010000000001000001010000?140?0000000?30000010010120012020010?0000000000000001010000033300000000000?200000100?00011000000200000013?0?13030???10100010200320[01]00010101000000[01]000?0000100011[01]00000010010001120111000000100000010011001010000000??2000?0000?001001101000??0?00?0?00100010

Anolis_carolinensis 10000?000200000?01?00001000010000??11000000?0?0?10000001120??0200000002?0?000110000?0?0100??1200000?01101000001113020000000000000100000000000001010120000010000000000002000?000010101200002000000000001000010??01?0000000??000?000000100000?0??010?0000001?0?21?101?0000?01?00000?0000?0000010?0?0100112100000000100000000?100?00?0000?0??00010100000?0101001001010000?140?2000010?3?000?1?1???21012020012000000?0000000001010000033300000000000?200000101?01011000000000000013?0?12020???101000[01]?[12]00[23]10100011101100001000[01]?[02]000100000100000000010001120111000000100000010011001000000000??2000?0000?0010011010000000000000010?010

Leiosaurus_catamarcensis 100000200200000?01000001000010000??10000000?0?0300000001120??0200000002?0?000110000?0?0101??0000000?01101000001111020000000000000100000000000001010020000020000000000001000?000010101200002100000000001000000??01?0000000??000?000000120000?0??010?0000001?0?01?111?0000?00100000?0000?0?00010?0?0000012100000000100000000?100?00?0000?0??00010100011001010000010100000130?2000010030000010010010012020010?00000?0000000001010000023200000000000?20000010??01000000??00000?00?2?0?12020?????1000??????????????1???0??0??00???00??0?0???0??00??00????11?011?0????0??0??????01?001??00???0???2000?0000?00?0?12????????????????10?01?

Pristidactylus_torquatus 10000?000200000?00000001000010000??10010000?0?0300000001120??0200000002?0?000110000?0?0100??0100000?01101000001112020000000000000100000000000001010020000120000000000001000?00001010120010?200000000001000000??01?0000000??000?000000110000?0??010?0000001?0?01?111?0000?00000000?0000?0?00010?0?0100012100000000100000000?100?00?0000????00010100000000010000010100000140?2000?10030000000010010011020010?00000?0000000001010000033200000000000?2000001010010[01]1000400000000012?0?12020?????1000100002100000111101000011000?100[01]10000[01]100000000010001120111000000100000010011001010000000??2000?0000?001001201000000?00?0?00100010

Urostrophus_vautieri 10000?000200000?01000001000010000??10000000?0?0400000001120??0200000002?0?000110000?0?0101??0000000?01101000001112020000000000000100000000000001010020000120000000000002000?000010101200102200000000001000000??01?0000000??000?000000110000?0??010?0000001?0?01?111?0000?00000000?0000?0?00010?0?01000121000000001000000000110?00?0000?2??00010100011000010000010100000140?2000010030000000010011012020010?00000?000000000100?000033200000000000?20000010??01000000??00000?00?2?0?1?010?????1000??????????????1???0??0??00???00??0?0???0??0???00????11?011?0????0??0??????01?001??00???0???2000?0000?00?0?12????????????????10?010

Aciprion_formosum 10000???0000?00?0000000??0001?000??10000000???0300000001120??1????000?2?0?0001?1000???0100??020?000?01101000?011?1020000000000000100000000000001000020?0002000?0?????000010?000?1?101??????00????0????????????????0????????????????????0???????0???001?00?????1??????000??0??0000?0000?0?00010?0???????2??0000????000000????????0?0000?????00????00?000???0??0??01?00001???100010002001?00001001201202?010?0000000000000001???0000?3?00000000000?2000??????????????????????????????????????????????????????????????????????????????????????????????????????????????????????????????????????1000?00????????????????????????????????

Crotaphytus_collaris 10000?000000000000000101100010000??10000000?0?0000000001120??1????000?2?0?000110000???0100??0000000?01101000001111020000000000000100000000000001010020000020000000000000100?000010101200102100000000001000000??01?0000000??000?000000110000?0??010?0000001?0?01?111?0000?00000000?0000?0000010?0?01001121000000001000000000100?00?0000?0??00010100000000010000010100000140?10?0110020010011010010012020110?000000000000000100?0000232200000?00000200000101001001000400000000012?0?13030???1010000?0002000001111101000011000?2000100000100000000010001120111000000100000010011001010000000??2000?0000?001001001000000?00?0?00100010

Gambelia_wislizenii 10000?000000000?00000101000010000??10010000?0?0000000001120??1????000?2?0?000110000???0100??0000000?0110100000111202000000000000010000000000000101002000002?000000000002100?000010101200202000000000001000000??01?0000000??000?000000110000?0??010?0000001?0?000111?0000?00000000?0000?0?00010?0?01000121000000001000000000100?00?0000?0??00010100010000010000010100000140?100001002001001001011101?020110?00000?00000000010100000333200000000000200000101001001000400000000012?0?12020?????1000100002000001111101021011000?2000100001100000000010001120111000000100000010011001010000000??0000?0000?0010010010000000000000010?010

Enyalioides_laticeps 100000200020100?00000001000011000??10000000?0?0200000001120??0200000002?0?000111000?0?0100??0200000?0110100000111101000000000000010000000000000100002000002?000000000000000?000011101200102200000000001000000??01?0000000??000?000000100000?0??010?0010001?0?11?131?0000?00000000?0000?0000010?0?0100112100000000100000000?100?00?0000?0??00010100000?01010000010100000140?0000000010010000010010012020012000000000000000010100000433000000000000200000101001011000000000000012?0?12020?????1000100002100000111001000011000?2000100000100000000010001120111000000100000000011001[01]10000000??20010110000110010????????????????10?010

Morunasaurus_annularis 100000200?20000?00?00101000011000??11000000?0?0200000001120??0200000002?0?000111010?0?0100??0200000?01101000001111010000000000000100000000000001?0002000002?0000?0000000000?000011101200102200000000001000000??01?0000000??000?000000110000?0??010?0010001?0?11?131?0000?00000001?0000?0000010?0?0100112100000000110000000?100?00?0000?0??00010100000001010000010100000140?0000000010020000010110012020012000000?000000000100?000033300000000000?200000101001011000000000000012?0?12020?????1000100002000001111111000011000?100110000000[01]0000100100011201110000001000000000110010[01]0000000??2000?0000?011001001000??00?000?0?10?01?

Brachylophus_fasciatus 100000000000000?00000001100010000??10000000?0?0?10000001120??0200000002?0?000111000?0?0100??0200000?011010000011130100000000000?0100000100000001200020000020000000000000000?000110101200102000000000001000000??01?0000000??000?000000111000?0??010?0010001?0?01?111?0000?00100000?0000?0?00010?0?0100012100000000100000000?100?00?0000?0??000101000[01]0?0[01]010000010100000140?10000000300000100100100110200120000101000000000100?0000232000000000000200000101001011000400000000013?0?13032?????10001000021000011[01]101[01]000011000?200010000000000000001000112011100000010000????0110011?0000000??2000?0000?0010010????????????????10?000

Armandisaurus_explorator ?00000100000000?00000001?00010?00??10000000?0?0200000001120??0200000002?0?000??1000?0?0100??000?000?0110200000111201000000000000010000010000000100002000002?000?00000000000?000110101200?0???????0?00?100?000??0???000000??000??00?0010?000?0??010?0010001?0?01?111?00?0?01??0000?0000?0?00010?0?010001??00000000?00000000?????00?00?0?????001?1000?0000??0??00?0100000140?100000003000001?0?0010011020012000000?0?????????01?000033?00000000000?2000?????????????????????????????1?0??????????????????????????????????????????????????????????????????????????????????????????????????????1000?00????????????????????????????????

Dipsosaurus_dorsalis 100000[12]00010000?00000001100010000??10000000?0?0200000001120??0200000002?0?000111000?0?0100??0000000?011020000011120100000000000?0100000100000001000020000020000000000000000?000110101200202100000000001000000??01?0000000??000?000000111000?0??210?0010001?0?01?111?0000?01??0001?0000?0000010?0?01000121000000001000000000100?00?0000?0??00010100010000010000010100000140?1000000?30000010010111011020012000000?000000000100?000023300000000000?200000101001011000000000000013?0?13002???101000100002000001111001000011000?2000110000000000010010001120111000000100000012011001010000000??1000?0000?0010010010000000000000010?000

Sauromalus_ater 100000[12]00000000?00000001000010000??10000000?0?0300000001120??0200000002?0?000111010?0?0100??0000000?0110100000101201000000000000010000000000000100002000002?000000000000000?00011010120010?100000000001000000??01?0000000??000?000000101000?0??010?0010001?0?01?111?0000?01??0000?0000?0?00010?0?01000121000000002000000000100?00?0000?0??00010100001001010000010100000130?0000000030000011010012011020112000010?000000000100?000023300000000000?200000101001000000100000000013?0?13002???1010000?000200000[01]101100000011000?200010000[01]10000000001000112011100000010000000?0110010?0000000??2000?0000?001001001000000?0000?0010?000

Liolaemus_bellii 10000?000000000?00000001100010000??10030000?0?0100000001121??0200000002?0?000112000?0?0100??0000000?0110100000011202000000000001010000000000000100002000012?000000000001100?000010101200?02200000000001000000??01?0000000??000?000000110000?0??010?0010001?0?11?131?0000?00000000?0000?0?00010?0?01000120000000001000000000100?00?0000?2??00010100010000010000010100000130?2001010030000010010211012020012000000?000000000100?000023200000000000?200000101001000000400000000012?0?11020?????100010[01]00200000011111100001100[01]0[12]0011000010000000000100011201110100001000000100110010000[01]0000??0000?0000?001001001000??0??000?0?100011

Phymaturus_palluma 100000200000000?00000001100010000??10000000?0?0100000001121??[01]?????00?2?0?000112020???0100??0000000?0110100000111202000000000000010000000000000100002000002?000000000001010?000010101200102100000000001000000??01?0000000??000?000000110000?0??010?0010001?0?01?131?0000?00000000?0000?0?00010?0?0100012100000000010000000?100?00?0000?0??00010100000?00010110010100000140?100101002000000001021001202011200000000000000001010000013300000000000?200000101001000000[014]00000000002?0?11020?????10001000020000001110[01]1000010000?200[01]10000010000000001[01]001120111000000100000011011001010000000??1000?0000?001001001000??0??000?0?10?011

Chalarodon_madagascariensis 100000100000000?00000001000010100??10000000?0?0200000001121??1?????00?2?0?000112000???0100??0000000?01101000000112020000000000000100000000000001000020000020000000000000000?000010101200002000000000001000000??01?0000000??000?000000110000?0??010?0010001?0?01?111?0000?00000000?0000?0?00010?0?01000121000000001000000000100?00?0000?0??00010100010001010?00010100000140?2000010030000000010111012020110?00000?0000000001010000033300000000000?200000100?01011000000000000012?0?11020?????10000?1004000001111001000011000?200010000100000000001000112011100000010000001?011001000000000??1000?0000?001001001000000?00?0?00100110

Oplurus_cyclurus 10000?000000000?00000001000010100??10000000?0?0400000001120??1?????00?2?0?000112000???0100??0000000?0110100000011202000000000000010000000000000101002000002?000000000002000?000010101200002000000000001000000??01?0000000??000?000000110000?0??010?0010001?0?00?111?0000?00000000?0000?0?00010?0?01000121000000001000000000100?00?0000?0??00010100010001010000010100000140?2000?10010010000010120012020110?00000?0000000001010000023200000000000?200000101001001000400000000012?0?11020???1010001000020000011[01]1[01]1[01]000011000?200[01]10000[01]00000000000000112011100000010000001[04]0110010[01]0000000??2000?0000?001001001000000?00?0?0010?110

Petrosaurus_mearnsi 100000100000000?01000101000011000??10000000?0?0000000001121??0200000002?0?000111010?0?0100??0000000?0110100000111202000000000000010000000000000100002000002?000000000000010?000010101200202200000000001000000??01?0000000??000?000000100000?0??010?0010001?0?01?121?0000?01??0000?0000?0?00010?0?01000121000000001100000000100?00?0000?0??0001010000000001001001010000?140?2000010030000010010121012020010?0000010000000001010000033300000000000?200000101101001000401000000002?0?11020???101000100002000000111001000011000?2000100000100000000010001120111000000100000010011001000000000??1000?0000?00100100100000000000?00100000

Uta_stansburiana 10000?000000000?00000001000011000??10000000?0?0000000001121??0200000002?0?000112000?0?0100??0000000?0110100000111202000000000000010000000000000100002000012?000000000000010?000010101200202200000000001000000??01?0000000??000?000000100000?0??010?0010001?0?01?121?0000?01??0000?0000?0000010?0?0100012100000000110000000?100?00?0000?0??0001010000000001000001010000?140?1000010020000010010121012020010?000000000000000100?000023300000000000?200000101001000000401000000002?0?11020???101000101002000000111011000011000?0000100000100000000010001120111000000100000010011001000000000??0000?0000?00100100100000000000?0010?000

Sceloporus_variabilis 10000?000000000?00000001000011000??10000000?0?0100000001120??0200000002?0?000111000?0?0100??0100000?01101000001112020000000000000100000000000001000020000120000000000000010?000010101200102200000000001000000??01?0000000??000?000000110000?0??010?0010001?0?01?131?0000?01??0000?0000?0000010?0?0100012100000000100000000?100?00?0000?0??00010100000?0001000001010000?140?2000010020000010010121012020010?0000010000000001010000033300000000000?200000101001001000401000000012?0?11020?????10001010030000011[01]101[01]000011000?00001000001000000000100011201110[01]00001000000100110010000[01]0000??1000?0000?0010010????????????????12??00

Phrynosoma_platyrhinos 100000100000000?00000??1100011000??10000000?0?0100000001120??1?????00?2?0?000111000???0100??02?0?01?001010000011110?000000010000010000001?000000?0002000122?000000??0010010?000010101200002400000000?010????????1?000000????00?00?000100000?0??010?0010001?0?21?121?0000?01??0000?0000?0000010?0?0001?120000000001000000000100?00?0000?0??0001010001100101000001010000?140?0000?000100100100102??010020210?00000?0000000001010000132200000000000?200000100?01000000001000010013?0?11030???10?000102002000000101000000011000?[02]000100000000000000010001120111002100100000010011001000004000??2000?0000?00200100100000000000?00100000

Uma_scoparia 100000[12]00000000?00000001100011000??10000000?0?0000000001121??1?????00?2?0?000112000???0100??0000000?0110100000111202000000000000010000001?000001?00120000120000000000010010?000010101200102100000000001000000??01?0000000??000?000000100000?0??010?0010001?0?01?121?0000?01??0000?0000?0000010?0?01000121000000001000000000100?00?0000?0??00010100000?0101000001010000?140?10000100300000100100110120202110000000000000000100?000023300000000000?200000101001011000401000000002?0?11020???101000101002000001111011000011000?0000100000100000000010001120111000000000000012011001000000000??0000?0000?00200100100000000000?0010?000

Leiocephalus_barahonensis 10000?000000000?00000001200010000??10010000?0?0200000001120??0200000002?0?000111000?0?0100??0000000?01101000001112020000000000000100000000000001000020000120000000000001000?000010101200102100000000001000000??01?0000000??000?000000110000?0??010?0010001?0?11?121?0000?01??0000?0000?0?00010?0?0100112100000000100000000?100?00?0000?0??00010100000?0101000001010000?140?2000020?3000001001012101202011200000010000000001010000033300000000000?200000101001011000400000000012?0?11020???1010001010030000011110[01]10000110010000010[01]000100000010010001120111000000100000010011001000000000??1000?0000?001001001000??0?00?0?0010?000

Plica_plica 101000100020?00?0000000?000011000??10000000?0?0300000001120??0200000002?0?000111000?0?0100??0000000?0110100000111202000000000000010000000000000100002000002?000000000001000?00001010120010?100000000001000000??01?0000000??000?000000111000?0??010?0000001?0?11?121?0000?00000000?0000?0000010?0?0100012100000000100000000?100?00?0000?0??00010100000?0101000001010000?140?2000020?3?00?0101???21010020110?0000000000000001010000023300000000000?200000101001011000100000000002?0?1?020?????10001000020000011[01]111[01]000011000?200000000010000000001000112011100000010000101[01]011001010000000??1000?0000?001001001000????00?0?00100000

Stenocercus_guentheri 100000100000000?01000001000010000??10000000?0?0300000001120??0200000002?0?000111000?0?0100??0000000?01101000001012020000000000000100000000000001000020000120000000000001010?000010101200002100000000001000000??01?0000000??000?000000110000?0??010?0000001?0?11?101?0000?00000000?0000?0?00010?0?01000121000000001000000000100?00?0000?0??00010100000?0101000001010000?140?20?0020030000010010121011020110?00000?000000000100?000023200000000000?200000101001001000400000000002?0?11020?????100010100200[01]0011111[01]10000110010100010000010000000001000112011100000010000001[02]011001000000000??1000?0000?0010010????????????????10???0

Uranoscodon_superciliosus 10000?000000000?00000001000011000??10000000?0?0300000001120??0200000002?0?000111000?0?0100??0000000?0110100000111202000000000000010000000000000000002000002?000000000000010?000010101200102100000000001000000??01?0000000??000?000000110000?0??010?0000001?0?01?121?0000?00000000?0000?0000010?0?01000121000000001000000000100?00?0000?2??0001010001000101000001010000?140?2000010030000010010121012020110?00000?000000000100?000023300000000000?2000001010010[01]1000100000000002?0?11030?????1000101002000001111111000011000?2000100000000000000010001120111000000100000010011001010000000??0000?0000?001001001000??0?00?0?0010?010

Tchingisaurus_multivagus 10000?0?0?0??00????????????0?00?0??0?010000?0?0?100000021?0??0010000000?0?00???00?1?0??1?0??000?000?01100000?010?30?0000000000000000000?00000001?00021000?20100???0000???0??00001?101200?0?????????????????????????0?0??????00?000???0?0?????????0??1?00??????????1???00?0?00??????????0???????0?0??0?????0000??1???0???????????0?0????????00??1??????0?????????????0001???0000000000030001010020011020010?0000000000000000010000033?000000?????02001???????????????????????001?0?1???????????????????????????????0????????????????????????????????????????????????????????????????????????2??0?000???????????????????????????????

Gobinatus_arenosus 10000?0?0000100?00?00??1?0000?000??010?0000?0?0?20000002110??0010000000?0?0001?00?1?0?1100??0104000?01100000?020?303?0000100000?0000000?0000000110000100002?100?00000010000?00101?101200?0??????????0????000???0????????0??000?000?????0????????10?00?00?2?0?0??1????000?000?0?01?0000?00000???0?0????1??0000?????000000????????0?0000?????0010??00???0?????00???100000140?000?000000030001010010011020010??001000100000000???0001?23000000??????2001?????????????????????00001?0?1????????????????????????????????????????????????????????????????????????????????????????????????????????1000?0000??????????????????????????????

Adamisaurus_magnidentatus 10100???00?0100?00?0000?000000000??01040000?0?0?100000021?0??0010000001?0?000100001?1?11?1??0004000?01100000002013010001010000000000000000000001100001000?10100000000010??0?00101?101?00?0???????0?00010?0001??0???0?0??0??000?000?0?100?00?0??010?0000002?0?01?000?0100?01??0101??000?010000??0?0000012?00000??1?000000????????0?0000?????0010?000?0?0???0??0??01?0100040?00?2?2000002000?01001001?020?10??00100010000?00000?040122100000000000?0001???????????????????????????0?1????????????????????????????????????????????????????????????????????????????????????????????????????????0000?0000??????????????????????????????

Gilmoreteius 10?00????0??00??0000?0???00000??0??000?0000?0?0?300?00021?0??0010000000?0?000100001?0?11?0??0004000?0110000001?01303?0000100000000?0?0000000?0011???010000101000000001??????????1?101100?0?????????0?010?000???????0100?????00?????0010??00?0??110??00000?????1?000??100?01??0101???00?0100?0??0?0?00?12?00000??????00?0????????0?0??0?0???0010???0?0?0???0??0??01?0000?40?0000?0?0000?100?01?0??01?0?0?10???0?0?010000?0000??000133300000000000?2000????????????????0200000?01?0??2?????????0?0???????0??????100?0210?1001100111??00??00000??00???????0???0?00000?000????1100010?00?000???1000?0000?0????????????????????????????

Polyglyphanodon_sternbergi 100000100000000?00?00001000000000??01040000?0?0?20000002110??0010000000?0?000100001?0?1101??0004000?01100000012013030000010000000000000000000001100001000010100?000001??????????1?101?00?0?????????0001010001??0???010??0??000?000?00120?00?0??110?000000?????1?001?0100?01??0101??000?010000??0?0??0??2?00000??????00?0??????????0000?????001????0?0?0???0??0?10100000040?0002?0000003100?01001001?0?0?1??0?01?0010000?00000?00013330?0000??????2000????????????????0200000001?0?12000??????0?????????0?0?01?100?0200??0010?0?????00??00000???01?00?1?0??10?00000?0000??????0??1?00?000???1000?0000?0????????????????????????????

Sineoamphisbaena_hexatabularis 10000????0???0??0000????000000??0??001????????????????011?0??00??0000???0?011?00????0?10?2??01140?0?1011?00????0??0??000010000??1??0?0??0????00?????01000010??0?0????1????????????011????0????11?????????????????????0??????0????????10???0????2?0??????1??0?01?13??1?0??01??02????000??100????0?????????00000????2???220????????????0??????1001??01100?????32?101?0000???????0???00?????????????0??0????10????0??00??0????010000142?0100?0??000?00????????????????????????0????0?1?0????????0?????????0????1?100???????0?????????????????????00???????????????????????????????????????????2000?0000??????????????????????????????

Adriosaurus_suessi 1?100???1???????????????????????0??0????????0???100?0?0?1???????0?00??0?0?00???0???????1?0??0?0?000??1??000???????0??1??????000?00?0?????????0????????????????0???0??????????????????????????????????????????????????????????????????????????????????????????????????????????0???????????????????0??0?1??00000??????????????????????????????????????????????????0??????????0?????????????????????????????????????????????????????????1????1??0???????????????????????0410003?11????3?30??????1?????????10??0??101?1?????1????0?????0???0?000??001??????0???0????0??011??????00????0??300???0??0?0?00??????????????????????????????

Pontosaurus 10100?0?1110000?0?11????0000?00????10????0?????400000001110??2??0?00000?0?0001?0??2???0100??0000000?0110000???0???2??1?00000000?00?0?0???????001??0?2000012??00?000010000?0?00001?101????1??????????0???????????????????????????0??????????????????????????????????????0?0????0????????????????0?0??0?10?0000???????????????????????????????????????????????????0??0????????000?0?00?0112??010?1201?0?0?111?0000??000000010?0?????2??100?01??????0??0????????????????0410002211?1??3?30???20?1?0??2???010???0??01?0??0??00???0?????0???01000??0011000?00???0?00001?011?????000012?0???00???0000?0?00?0????????????????????????????

Aigialosaurus_dalmaticus ??????????????????11?????????0?????1???????????400000?0?110??2??0?00??0?0?0001?0??3???01?0??020?0???0110000???????01?1?0?0?0000?00?0?0??00???0?1??0?200??12??00???00?????????0????101????1???????????????????????????????????????????????????????????????????????????????????????????????????????0????????????????????????????????????????????????????0?????????????01?2???0????00??00???????10???1?0??????????0???0??????????00?1???1?0??1??????0???????????????????0400000211010???30???20?1???????????????????????????????0?????????010?0??002??????0???0??0000?0????????00????0????0???0000?0?00?0????????????????????????????

Clidastes 1110012?021?000???11?????1???0?????100?0000???0?1110000??10??2??0?00??0?0?000300003???1100??0202000?011000003?0???01?10000010000200000??000000010000200001??0?0?00000000000?000010101200?1???????0?????????????????????????????????????????????01???0???0?????1?10??00?0?001?0?00????2?00???2??0?0?00??0?000000?10000010001?????0?0000?0???010?1000?00010?0131?101?001?2???0000?000000?12??01101??10?202?11?0?01??00??????000?000222210000111001?0000????????????????041000021001013?30??????0?????????10??01?????1?????0????0?0?0?00??01201??101?1???00???0???100?011??????00????01??10???0000?0000?0?0??????????????????????????

Platecarpus 1110012?021?000???11?????????0?????100?0000???0??1100?0??10??2??0?000?0?0?001300003???1100??1202000?011000003?0???01?10000010000200000??000?000100002000012?0?0000000000000?000010101200?12???0??0??????????????????????????0??0?????1??0??????010??00000?????1??0???0????01?0?00??002?000002??0?0?00?10?000000?10100010101????00?0000?????00??1100100010?0131010100012241?00?0?0?0000?120?0?10120101202011?1?00?000????0?000?0002223010001?000110000????????????????040000021001013?30???20?1??????????0???1?101?1?????1??????????????012?1??101?????00????????0??011??????00??2?0???10???0100?0000?0?31?????????????????????????

Plotosaurus 1?10012?0210000???110????100?0?????100?0000???0?0110000??10??2??0?00??0?0?000300003???11?0??0202000?011000003?00??0101000001000?200000000000000100?02000012?010?00000000000?0000?0101200?1?00??????00010?000???1???0010?0??000?00000??10000????010?0000002?0?01?10??001??001?0?11??0?2?000202??0?0??1?10?000000???0000?0????????0?0000?0???010?1000?000?????3???0101012241?0000?0000001120001101201012010110?001?000100001000?00022322100011000110000????????????????042000021?01010?30???????????????????????????0000??0??????????????012?1??101?????00???1???100????????????????????1????0000?0000?0????????????????????????????

Tylosaurus 1110012?021?000???11?????1???0?????100?0000???0?01100?0??10??2??0?00??0?0?001300003???1100??0202000?011000003?0???01?1000001000?200000??000??011?0002000012?0?0?00000000000?000010101200?1????????????????00????????????0??????????????????????01???0???0?????1?10??00?0?00100?00??002?00???2??0?0?00??0?000000?????????00???????????0????????????????01??0?3??????0012241?0000?000000?12??01101?0100202?11???00??00?10001000?0002222100001?000110000????????????????040000021001010?30???30?0?0???????100101?001?1??????????0?????0???01201??101?1???00???0???100?011??????00??2?00??10???0?00?0000?0?11?????????????????????????

Eichstaettisaurus 0?0?0?0?00?000??01000?0??000?0000??001?00?0?0??10000??01?10??00?0000000?0?0001?00?2?0?00?2??0?0?000?01100000??0??301?0?00000000?00?0????00??0001000?2000002?000?11001001000?00001??012?0?0??????????????????????????????????????????????????????2????0???????0???3???0?0?0???0?10?0?00?0???02??000?00?1??00000??????????????????????????????????????????????????0?00000?40?0?0??0000?03????01??1?01?020?1????000??00????0?000?000044?0000?0??0???0?00????????????????0400000002?0??1020??????0?????????00?????????0??1???????000???0???00000??001??0??10???0?00001?000?????1100???00?000???0000?0000?0????????????????????????????

AMNH_FR_21444 ??????????????????0????????0?0000??00130?00?0?0300000001010??00?0000000?0?00???0012???0002??0?1?000?01100000?0?1??0???000000000200?00?1???000001?00020?00?2??1???????000??0????0?????????????????0??????????????????00?????????????0??00??????00???000?002?0?01?131?00?0?01??0010?0000?0?010200??0??0????0000?0?1001100????????03?000??2???0010100????0???1??0??01??????????00????00002??00010010?1?020?1????0?0??00??????0???0??0????00?00????????????????????????????????????????????????????????????????????????????????????????????????????????????????????????????????????????????????0000?0000??????????????????????????????

Delma_borea 100000100100100?00000120000000000??1033000000?0?20000101410??0000?01?1?????????????????002??0210000?11?1?002000013010000000100032000001?1?001010?00010?2????011??????1??00??00???2101200401300000000001100011??01?0000000??100?000000100010?1??030?0000002?0?01?121?0000?01??0011??000?0??2?21?0?000011200000000101000010??0???00?0000?0??00000100000?0?01200001010000?230?2000020030000?001???0?0100200110000001000000000000?0001?3?10000100000??0000110??01000???????????00???0?1?020??????01???????????????????0?????1??????????????????????????????????????????????????????????????????0000?0000?0011?11????????????????10???0

Lialis_burtonis 10010?000100000?02?001100000?0000??1133000100?0??00001011?0??0000?01?0?????????????????102??0210000?01?1?000000014010000000000032000001?1?0011??????????????0?1??????1??00??00???2101200401100000000001100011??01?0000000??100?00000010?010?1??030?000001200?01?121?0000?01??0010?3000?0??2?20?0?000001200000000101000000?00???00?0110?0??00000100000?0?01200001010100?240?2000020030000?001???0?01002001100001010000000000010000044410000100000?000001101001000000000420030011?0?10020??????0100?31??0110000?00?10001111????0?????0?2?01001??01???????????????????0111112?????????????????0000?0000?00010110?00100011010201100??0

Strophurus_ciliaris 10000?000000000?0000?000000010000??1033000300?0300000001020??0000?01?1?????????????????002??0010000?01?10002001013010000000000031000001?1?001001?00020?2????01???????????????????2101200401201000000001100010??01?0000000??000?000000100010?0??030?0000002?0?01?131?0000?01??0011?2000?0??2?21?0?001001200000000100110000??0???03?0000?2??0001010000100?01200001010000?240?0000010?30010?001???3101002001200000010000000000010000044400000000000??00?00101101000000200200000?02???01020?????10000?100200100011100102101101??10011100000010001?0010001120111010000100000001110001000010000??0000?0000?0001011010010001?01000?100??0

Rhacodactylus_auriculatus 10000?000000100?01?00100000000000??1132000300?0?110000011?0??0000?01?2?????????????????002??0?10000?01?1?000001014010000000000030000001?1?001001?00000?2????011??????002000?0000?2101200202300001000001100010??01?0000000??000?000000100010?0??030?0000002?1?01?131?0000?01??0011?1000?0??2?21?0?001001200000000100120000?00???03?0000?2??0001010000100?01200001010000?240?0000010030010?001???3101002001100?0001000000010000?00003330000000000??000001101101000000200200000002?0?00020???0?10100?1002?000001?100?021011001120001100010000001?001000112011001000010000000[024]111001000000000??0000?0000?00010110100100011010?01100??0

Saltuarius_cornutus 000000200020100?00000110000000000??1033000300?0301000001110??0000?01?2?????????????????002??0210000?01?1?000001?14010000000000030000001?1?001001?00120?2????001??????002000?0000?210120040?300001000001100010??01?0000100??000?000000100010?0??030?0000002?1?01?131?0000?01??00?1?000??0??2?20?0?001001200000000100120000??0???03?0000?2??00010100001?0?01200001010000?240?0000010?30000?0?1???0?01002001200000010000000100010000044400000000000?00000[01]101101000000200200000002?0?0?020?????10[01]00?20[01]300000010110002101101??10001000010010001?0010001120110000000110000000110001000000001??2000?0000?0001011?????????1??????100??0

Aeluroscalobates_felinus 100000200000100?00?00000000000000??1131000100?0300000001110??0000?01?1??0??????????????102??0000000?0111?000000013010100000000040000001?1?001001?00000?2????010011001020020?00001210120040?201000000001110011??11?0010100??000?010000100010?0??030?000000221?01?131?0000?01??0011?0000?0??2?20?0?001001200000000100120000000???00?0000?2??00010100000?0001100011010000?240?0000010?300000?001003101002001100000011000000000010000044400000000000?000001101[01]01000010200200000012?0?11020???0?10000?2002001000121010001011001020001010010010001?0010[01]01120110000000100000000[01]10001000000000??0000?0000?00010110100100011010001100??0

Coleonyx_variegatus 10000?000000100000?00000000010000??1131000000?0300000001110??0000?01?1??0??????????????102??0010000?01?1?000000013010000000100040000001?02001001?00010?2????010??100?1???????????2101200402401000000001100011??11?0010000??000?010000100010?0??030?0000002?1?01?131?0000?01??0010?0000?0??2?20?0?001001200000000100110000000???00?0000?2??0001010000100001200001010000?240?0000010?30000??01???31010020011000000?0000000100010000144400000000000?0000001011010000102?0200000002???11020???0?10100?100200100012101002101100111000101002?010001?0010001120110010000100000000111001000010000??0000?0000?000?0100100100011010001100??0

Eublepharis_macularius 10000?000000100?00?00010000000000??1131000000?0300000001110??0000?01?1??0??????????????102??0000000?0111?000000013010000000000040000001?02001001000010?2????010011001020020?000012101200402301000000001100011??11?0010000??000?010000100010?0??030?0000002?1?01?131?0000?01??0010?0000?0??2?20?0?001001200000000100110000000???03?0000?2??0001010000100001200001010000?240?0000010?30000??0010031010020011000000?0000000010010000144400000000000?000001101101000010200200000002?0?11020???0?10100?100200[01]00010100102[01]?110011000111[01]0010010001?0010001120110000000100000000111001000000000??0000?0000?00000100100100011010001100000

Teratoscincus_przewalskii 10000?000010000?01000100000000000??0031000200?0300000001110??0000?01?1?????????????????002??0000000?0111?000000013010000000000030000001?1?001000?0?010??????011??????1???????????2101200?023000000000011000011??1?0010100??000?010000100010?0??030?0000002?0?21?131?0000?01??0010?0000?0??2?20?0?001001200000000100110000??0???03?0000?2??000101000010??01200001010000?240?000001003000000101003101002001100000000000000100010000043300000000000?00000010??011001102?0200000002?0?01020???0?10000?????000000101?0?0210110011100011001[01]0010001?0020?011?011?0?00001?0000?0?1110011?00?0000??2?0100000?0011?10010010001?01000?100??0

Gonatodes_albogularis 10000?000110000?01?00100000000000??1132000200?0400000001110??0000?01?1?????????????????102??0011000?01?1?000000013010000000100030000001?1?000000?00010?2????01???????????????????210120030?301000000001100011??11?0000000??000?000000100010?0??030?000001201?21?131?0000?01??0111?0000?0??2?21?0?0000012000000001001000000?1?0?03?0000?2??00010100001?0001100001010000?240?00010200300?0?0?1???3101002001100000?10000000100010000143300000000000?000001101101000000200200000002???01020?????10000?10020000001010000000110011100011[01]002?010001?001000112011001000010000000[03]110001000010000??0000?0000?0010012010010001101000110?000

Phelsuma_lineata 10000?000000000?12?00120000000000??1132000100?0?10000001110??0000?01?1?????????????????102??0010000?01?1?000000014010000000000012000001?1?001000?00000?2????011??????000000?0000?2101200302301000000001100011??01?0000000??000?010000100010?0???30?000001201?21?131?0000?01??0011?0000?0??2?21?0?000001200000000100100000000???03?0000?2??00010100001?0001200001010000?240?0000000030000?001?????01002001200000010000000110010000143300000000000?000000101101000000200200000002?0?01020???0?10000?100200000011110102101101??101[01]100001000000000010001120110000000010000000110001000000001??0000?0000?001[01]0100100100011010001100??0

Gekko_gecko 10000?000000100?02?00100000000000??1131000200?0300000001110??0000?01?1?????????????????102??0010000?01?1?000000013010000000000032000001?1?001001?00020?2????011??????001000?0000?2101200202200001000001100011??11?0210100??000?010000100010?0??030?0000002?1?21?131?0000?01??0011?2000?0??2?21?0?0010012000000001001200000?0???00?0000?2??00010100001?0001200001010000?240?0000010030000?001???3101002001200000010000000110010000044400000000000?0000001011010?0000200200000002?0?01020???0?10100?[12]00[23]00000010100?021011001110001100000000001?00100011201100[01]0000100000?0?110001[01]000000000?000101110000110110100100011010001100000

Lacerta_viridis 10000?000000100?02?00111000000000??01040000?0?0?10000002100??00001300?0?0?001100002?0?0112??0000000?01100000000014011000001000010000000000000000100020000110100001001000010?0000101011001022000000000011100011?21?0010101??102?000000100000?0??020?1100003?0?11?111?0000?00000011?2000?0?00020?0?01000121000000010000000001110?00?0000?0??00010100000?0001000001010000?240?0000010000030001010110011020012000000000000000000100000333000000000000100000101001000000300400000002???11000???001000101002001000111001021111001110110010000000000000100011101110[01]0000100000000110001000000000??310101000000100100312201100010201101000

Takydromus_ocellatus 100000100000000?02?00121000000000??01010000?0?0?10000001400??2????300???0?001100002???0112??0000000?01100000000013011000001000020000000003000000100020000110100001001000010?0000101011002023000000000011100011?21?0000101??102?000000100000?0??020?0100003?0?11?121?0000?00000010?2000?0?00020?0?0100012100000001000000000?110?00?0000?0??00010100000?0001000001010000?240?0000010000020001010111011020012000000?000000000000?0000433000000000000100001101102000000300200000?02???11020???0?100010[01]00[23]0000001110010211110011101110100[01]0000000000100011[01]011[01]0[01]000010000000[01]110001010000000??110101000000100100312301100010201101000

Colobosaura_modesta 100110100120100?00?00121000010000??11020000?0?0?20000000?00??00001001?0?0?010100012?0?0112??0100000?0111?002000013011000000000030000001003000000?00010000110100011001001000?0000111011001021000000000011100010?01?0010101??102?001000110000?0??020?000001301?11?111?0000?00??0010?2000?00?2?20?0?0000012100000001000000001?110?00?0000?1??00011101?00?0001000001010000?240?100001000002000101023101002001200001010100000000010000043300000000000?1000021???020001110?0200000102?0?13002?????10001?1???????????11??0???????1??01????0???0??0???00????11?0???0???????0??????11?001??00???0???2000?0000?00?0?10041231110?01020?11????

Pholidobolus_montium 100110100120100?01?00120000000000??1133000000?0300000000?00??00001101?0?0?000100022?0?0111??0100000?0111?002000013011000001000031000001003000000?00000000110100001001001000?0000111011001021000000000011100110?01?0010101??102?001000110000?0??020?000001300?11?111?0000?01??0010?2000?0001020?0?0100012100000001000000001?110?00?0000?0??00010101?0000001000001010000?240?100001003000000001023101102001200001010100000000010010042200000000000?100001111002000111000100000102?0?13002?????100010100210000[01]10111002111100111011101000001000000011101110111000000100000000110001010000000??0000?0000?0010010041221110?01020?11?110

Callopistes_maculatus 10000?000000000?00000101000010000??10000000?0?0300000002110??0000100001?0?000200012?0?1110??1200000?0111?00000001301100000000003000000000000000010002000012?100001001000000?0000111011002021000010000011100010?01?0010101??102?000000110100?0??020?0000003?0?11?101?1000?00100011?2000?0101020?0?01001121000000010000000011100?00?0000?0??0001110001000101000001010000?140?0000100000030000010110011020011000010?0100000000110010132300000000000?100001111001000111000100000012?0?13002???0?10001?101200000010110000001000100010111000000000010011101120111000000100000001110001010000000??0000?0000?00100100412011100010201101110

Tupinambis_teguixin 10000?000000000001?00111000010000??11000000?0?0300000002410??0000100002?0?000200012?0?1110??1200000?0111?00010001401100000000003000000000000000010002000012?100001001000000?0000111011001020000010000011100010?01?0010101??102?000000100100?0??020?0000003?0?11?101?1000?01??0111?2000?0101020?0?0000112100000001000000001?100?00?0000?0??00011100000?0101011001010010?140?000001000003000001011001102001200001010100000000110010043220000000000?2000022110011??111000100000012?0?13002???0?10000?10020000001011[01]001111100[01]0[12]0[01]011100010000000001[01]10112011100000010000000?1100010?0000000??2000?0000?00100100412011100010201101110

Aspidoscelis_tigris 10000?000000100?01000100000010000??10000000?0?0300000002110??2??0?000?0?0?000200012???0110??0003000?0111?00010001401100000000003010000000000000010012000012?100000000000000?0000111011001022000000000011100010?01?0010111??102?000000110100?0??020?0000003?0?11?101?1000?00100111?2000?0101020?0?01001121000000010000000011100?00?0000?0??0001110000000001000001010000?140?000010000001000101021001102001200001010100000000110000133300000000000?100001111101000111000200000012?0?13002???0?100010100200000110110102101100101000111000100000010011101110111000000100000001110001010000000??2000?0000?00100100402010100010301101??0

Teius_teyou 10000?000000100?00000100000010000??10000000?0?0400000002110??0010100000?0?000200012?0?1110??0003000?0111?00010001401100000000002010000000000000010002000002?100000000000000?0000111011001022000000000011100010?01?0110101??102?000000120100?0??020?0000003?0?11?101?1000?00100111?2000?0101020?0?0100112100000001000000001?100?00?0000?0??00011100001?0001010001010100?140?00001000000300010102100110200120000100010000000010?0000222000000?0000?100001111001000101000200000012?0?13002???0?100010100200000011110102101100101000111000[01]000000[01]00111011[12]011100000010000000111[01]00101000?000??1000?0000?00100100402010100010301101110

Paramacellodus 100?00200000000?01?001?00000??000??0?010000?0?0??00000000??????????????????????????????1?2??001000??011000?0?0001301100000000001000000?0?????00??00000000?1000?????0??????????????????0????????????0???????????????0?0??????????0????????????????0?011?003?0?100?????0?0?000?0?10?2000?000102??0?0????1?????????????????????????0?0???????????????????????????????000??240?00?1?0000002?000??0011?????0?1????0?????0??????00100000332000000?0000?2000???????????????????????????0??????????????????????????????????????????????????????????????????????????????????????????????????0???????2??????????????????????????????????????

Parmeosaurus_scutatus 1000001?0000000?0??00??0000000000??01010000?0?0?3000000?110??0010000000?0?00???00?2?0??100??000?100?01100000?00??301?000000000010000000??????001?00020000???10???????0??0?0?0???1?101200?0???????0?00?11?000???2???0?0??1??100?000??01?0000??????0??100003?0?1001???0000?000?0?10?2000?000102??0?0?00?12?00000????00?000????????0?0000?????001?1?00?0?0???0??0??01??000230?00010000000300000100?101?020?10?0001000000000010010000033300000000000020?0???????????????????????0?2?0?1???0??????0?????????00????????????????????0?????0???01000??00?0?????????????????0?0????1??????????????1021020221?010???????????????????????????

Tepexisaurus_tepexii 10000???0????????????????????????????0?0?0????0?4?0???0?1????2????000?0?0?02?1?00?2??????2???????????11??00???0???????0000100????????????????00???0??00001???000??0?1020?20?00001??01200?0??????????????????????????????????0??????????????????0?0?????0?3?0?11??3???00???1??0?11?2000?00100???0?0??0??????????????10????????????????0????????0???0???0???????????00000240?00020000200000?001001001?02001????010?010?????000??000?4430000000?000?2000????????????????0200000002???1?0????????0?0???????000?01?1???0??1??0????0?????0???0??00???010101?10???0?00001?00??????110010?00?000??????0?0000?0????????????????????????????

Cricosaura_typica 10000?000200000??200012??00010000??1133000000?0?30000001110??2??0??00?0???021100012???0102??0000001?11110002000013011000000000011000001?1?002020?0000000012?00000100102?020?0000101012102023000000000011100010?21?0200101??000?000000110000?0??120?1000003?0?11?131?0000?01??0111?3000?0010020?0?0100012100000001011000000?0???00?0100?2??00000100001?00110?0001010000?240?0?02020?302?1???1???11010010?10?1001010000000000000000132300000000000?000001101101010000300200000002?0?10000???1?10000?100200000011100101111100101011001002?010000000100011101110120001000000001100010[01]0010000??2000?0000?001001001122??1?1010?0?101??0

Lepidophyma_flavimaculatum 10000?000000000?02000101200000000??01040000?0?0??1100002110??2??0??00?0???020200002???0012??0010001?11110002000013001002001000010000001?1?001000?0000000111000001121102?020?0000101011102024000000000011100110?21?0200101??000?000001110000?0??220?0000003?0?11?121?0000?01??0211?3000?0010020?0?00000121000100010110000?01110?00?0310?2??0001010100000011000001010000?240?0002020?302?1??11???10010020110?1001010100000000010000032200000000000?200001101101010000300200000002?0?10000???1?10000?11??0000001110[01]10210110010[12]011101002?010000000101011[12]0111010000100000000110001000010000??000??0000?00100100112201101010?01101001

Palaeoxantusia_sp. 10000?0?0010000?01?00????00000000??01030000?0?0?30000001110??2????000?0?0?020200002???0002??001?001?1111?00??000?3011000001000010000001?1?001020?00000001110000011211?20020?00??1?1012?000??????????0????100???2???1????1??000?00????1?0000????220?1000003?0?11?1?1??000?01??0111?3000?001002??0?0??0?12?000100????100??????????0?0000?????001010?0?1?0?1?0??0010100000240?0002020?302010001???10010020110?100101010??0??00010000232200000000000?2000????????????????020??00002?0?1?0????????0?????????0?????????????????????0?????0???01??0???????????????????????????????????????????????3000?0000?0????????????????????????????

Xantusia_vigilis 10000?020010000001000121100000000??01030000?0?0?20000001010??2000000000?0?020?00022???0002??0000001?11100002000013011000000000010000001?1?001020?0000000012?00001101102?020?00001010121040?300000000001111001??21?0200101??000?000001110000?0??120?1000003?0?11?131?0000?01??0111?3000?0010020?0?010001210001000101100000010???00?0100?2??000001?000?000110?0001010000?240?0002020?302?1??01???11010020110?1001010000000000010000232200000000000?000001101101010000200200000002?0?10000???1?10000?11??00000011101102111100101011001002?01000000010101110111000000100000000110001010000000??2000?0000?00100110112201101010?01101001

Platysaurus_imperator 100001220000000?01?00100000000000??01030000?0?0300000001110??000000000??0?021200022?0?0102??0010201?111000010000140110000010000100000000?2000000?00000000110100011201020020?0000101012002023000000000011100110?11?0010101??100?000000100000?0??030?1100003?0?11?131?0000?01??0011?2000?0001020?0?010001210001000100000000?1110?00?0000?0??00010100000?0?110000010100000240?00020200000100?0010111010020010?1000010001100010010000033300000000000?00000110??01000000010200000102?0?11010???1010?00?00020000001110?100011100101001001001000000000010001?101110?00001?000????1100??0?00?000?1?200101200010?00100111201100010001??1??0

Cordylus_mossambicus 100001200020000?02000120000000000??10030000?0?0400000001110??020000000??0?021200022?0?0102??0010201?11110002001014011000001000010000000002000000?00000000110100011201020020?0000101012002022000000000011100111?11?0010101??100?000000100000?0??030?1100003?0?11?131?0000?01??0011?2000?0000020?0?000001210001000100000000??110?00?0000?0??00000100000?0?110000010100000240?00020200000200?0010111010020010?0000010001100010010000033300000000000?20000110??01000000??02000?0002?0?11010???1010000?00020000001110?1000111001010111010010010000000100011201110?00001?000????1100010?00?000?1020010121101010?100111201100010001101??1

Zonosaurus_ornatus 100000100000100?01000120000000000??00030000?0?0?30000001110??0?000000???0?021100012?0?0102??0110202?11100001000014011000001000010000000002000000200000000110100011201020020?0000101012001023000010000011100010?21?0010101??100?000000110000?0??020?0100003?0?11?131?0000?00000011?2000?0?01020?0?0000012100010001000000000?110?00?0010?0??00010100000?0111000001010000?240?000201000002000001012001002001101001010000000010010000033200000000000?100001101001[01]?0000000400000002?0?11010???1010000?1012000000111001021111001110111010011010000000100011[12]011100200010000000111000101000000010?101?2212010100100112201100010001101??0

Cordylosaurus_subtesselatus 100000100000000?01?00120000000000??00030000?0?0400000001010??00000000???0?021100012?0?0102??0110202?11110000?0001401100000010003000000000200000020000000?11010001120?020020?000010101200?023000000000011100010?21?0010101??100?000000100000?0??020?0100003?1?11?131?0000?00000011?2000?0??2?21?0?010001210001000100000000?1110?00?0010?0??0001010001000?11000001010000?240?00020100000100000101110100201110200?01000000001000?000133300000000000?00000120??011?00001?0400000?02?0?1?002?????10?0???????0??????1???0??????0???0?????0???0??00??00????11?0???0???????0??????11?001??00???0?10?101?2222010?0000011220110001000?101??0

Myrmecodaptria_microphagosa 0000012?0000000?0??00?00?0000???0??11010010?0?03000?00011?0??2????10000?0?00?1?00?2????1?2??000?000?01100002?000?301?000001000?1000?00??1????001?000000000200?01010010010?0?00001?10??0??0?????????????????????????????????????????????0???????0????1?????????1?1?1?0000?01??0?10?1000?0001?2??0?0?????2??0000????????????????????0000?????0010??0??????????????0?00100?40?0?0201000001?0?001001101?020?10??000010000000000???0000???000?0???????00?0?????????????????????????????????????????????????????????????????????????????????????????????????????????????????????????????????????02????????0?????????????????????????????

Carusia_intermedia 0000012?0000000?01?00000000000000??11010010?0?010000000?1?0??2????10000?0?0001?0022???01?2??0200000?011000020000?301?00000100001000000001?000001?00000000010000101001???????????1?101?0??0??????????0????000????????????1??????0?0?????00????????0??1?0003?0?2???3???000?0???0?10??000?000102??0?0?0??12?00000???????0??????????0?0000?????001???00???0????????1??000??2???00?2?1000?0200??0?011101?020?1????00?1010???0?000??0000433?00000?0?????000???????????????????????0???0?1????????????????????????????????????????????????????????????????????????????????????????????????????????3000?00??0?????????????????????????????

Globaura_venusta 000000200000000?01?00120000010000??11010000?0?03000000021?0??0000120000?0?0101?00?2?0??1?2??011?100?01100000?000?301?0000011000200000000????0001?00000000011000101001020020?000010101200?0?????????????????????????0?0?????100?000?2?100000????020?01000?3?0?11?131?0000?00000010?1000?0001020?0?0?00??2?00000????0000??????????0?0000?????00101?00???0???0??0??01??0002?0?00?20000000200010100?001?020?100000101000000101000?000123?0000000000002000??????????????????????????????????????????????????????????????????????????????????????????????????????????????????????????????????????2000?00????????????????????????????????

Hymenosaurus_clarki ?????????????????????????????0??0??0?0?000??0?0?4?0?000???0??0000120?00?0??????0??2????1?2??011?100?01100000?0?0?301?00000110???10?00????????001?00000000?11000?010010????????0010101200?0????????????????????????????????????????????????????????????????????10?3???000?000?0010??000?000102??0?0100?1??000???????000?1????????0?000??????001???00?1??0110?????01000?0240?00???00000010001010121011000010?0001010000001010???0001??2000?0???????2000??????????????????????????????????????????????????????????????????????????????????????????????????????????????????????????????????????2??????????????????????????????????????

Eoxanta_lacertifrons 0?000?0?0000000?01?00121000010000??01110000?0?0?2?000002110??0000120000?0?01?1?00?2?0?0102??0000000?0110000?0000130110000011000200000000020000012000000000110001010?1???0???00??1??0?200002??????0?00011?0001??2??00?0??1??100?000?20??0000?0??120?010001300?11?131??000?01??0011?1000?000102??0?0??0?12?000000???0000??00??????0?000??????00101000?1000110?00?101?0000240?00020000000100010100110?1020010?00010?000???0?1000?000122200000000000?2000???????????????????????00????1??????????0?????????0???????????????????????????????????????????????????????????????????????????????????2?0????????????????????????????????????

Plestiodon_fasciatus 00000?000000000002?00120000000000??01020000?0?0?20000001110??0000130100?0?011100022?0?0102??0110100?01100002000013011000001000020000000002000001200000000110100111001020020?000011101200?023000000000011100010?21?0010101??100?000020110000?0??020?010002300?11?131?0000?00000010?1001?0?01020?0?0000012100000001001000000?110?00?0000????0001010001000011000001010000?240?00010100000200?0010011011020010?00010110000000100100000333000000000000000001101001000000100200000002?0?11000???2010001?100210000011101102111100111011001002?010001?001000111011101000010000000011000101001000010210202222110100100112101000011001100??0

Scincus_ ?02000[12]000?0000?02?00101000000000??01020000?0?0400000001110??20?01201?0?0?010100022???0102??0110100?01100002000013011000001000021000000002000001200010000010100111001020020?0000111012001021000000000011100010?21?0010101??100?000000110000?0??220?010002300?01?131?0000?00000010?1001?0?01020?0?00000121000000010000000001110?00?0000?1??0001010001000011000001010100?240?00010100000100?0010011011000010?0001010000000010100000123200000000000?00000110??01000000100300000102?0?11000?????10000?101210000011100102101100111011001002?000001?0010[01]011201110102300000000001100010100[01]?20010210202222110100100112001000011001100000

Brachymeles_gracilis 00000?000000000?02?00120000000000??01030000?0?0??0000001110??2??0?201???0?0?0100022???0101??0110100?01110002000014011000001000022000000002000000?00000000110?00111001020020?0000111012002022000000000011100010?21?0010101??100?000020110000?0??120?010002310?11?131?0000?01??0010?1001?0??2?20?0?000001210000000100000000011?0?00?0000?1??0001010000000001010001010000?240?00010200000100?0010021011020010?0001011000000010010000133300000000000?000001101001000000100400000102?0?10010???2010000?100[234]11000011101102111100111011001002?010001?00000011201110011000000000001100010100[01]2000102102022221102001001120010??011?0?100???

Acontias_percivali 000100100000000?01?00100000000000??01230000?0?0??1000001110??00001?1?0?????????????????101??0210000?11100002000013011000?00000032000000002000020?????0?0012?0?00?1?01020020?0000111112000022001102000011100011?21?0010111???00?000020120000?0??220?010002310??1?131?0000?01??0011?1001?0??2?21?0?010011210000000102??0120010?0?00?01?0?2??0011010??01000110?0101010000?240?00010200300000?0010011010000010?00010?100000001000?000122200000000000?00000100??001???????0430030111?0?10000??????001??????02??????????1?????1????0?????0???0?000???1???????????????????1?????????????????????10000202222110?0?100112011000011001100??1

Amphiglossus_splendidus 000000100000000?02?00120000000000??01030000?0?0400000001110??0000130100?0?010100022?0?0101??0110100?01100002000014011000001000032000000002000000?00000000110?00111001020020?0000111012001022000000000011100010?21?0110101??100?00002?100000?0??120?010002310?11?131?0000?01??0010?1001?0??2?20?0?00000121000000010000000001110?00?0000?0??0001010001000011001001010000?240?00010200000100?0010021011020010?00010?1000000010010000133300000000000?000001101001000000300410000?02???1?000?????1000[01]?101210000010101002111100111011101002?010000000100011101110030001000000001100010100000001021020222211010010????????????????100???

Feylinia_polylepis 100000100000000?12?00100000100000??01020000?0?0??00001011?0??0000????0?????????????????100??0110100?01110002000014011000?0?10003200000001?0001????????????????01?1001020020?0000111112001023001112000011100010?21?0110111??100?00002012?000?0???20?010003310??1?131?1000?01??0111??1???0????20?0?010101210000000101000110??0???00?01?0?1??0001010?00000?0100?101010000?240?00010200000100?0010111010020110?000101100000001000?000222210000100000?00000[12]10??001??0????0430000211?0?1?001??????001??????31?1?0??00??0000201????1?????1???0100????1???????????????????1?????????????????????10100202222110??110????????????????10????

Trachylepis_quinquetaeniata 00000?000000000?02?00100000000000??11010000?0?0300000001110??2??0?301???0?011100022???0102??0110100?01100002000013011000001000030000000002000000?00000000010100111001020020?000011101200?02200000000001110001??21?0210101??100?000020110000?0??120?010003320??1?131?0000?01??0010?1001?0??2?20?0?01000121000000010000000001110?00?0000?0??00010100000?0011002001010100?240?00010100300100?0010020011020010?000101100000001000?000033300000000000?000001101101000000300200000102?0?11000???2010000?101200000111100102111100111011001002?01000000010?011201110?00001000000011100011100?0000102002022221101001001120010000110011?0??0

Sphenomorphus_solomonis 00000?000000000?01?00120000000000??11110000?0?0?30000001110??0000130100?0?010100022?0?0102??0110100?01100002000014011000001000032000000002000000?00000000110100111001020020?0000101012002022000000000011100010?21?0210101??100?000020120000?0??220?010003320??1?121?0000?01??0010?1001?0??2?20?0?000001210000000100000000??110?00?0000????00011100001?0?11002001010000?240?00010200300000?0010111011020010?0001010000000010010000133300000000000?000001201001000000300200000202?0?1?000?????100010101211000111101102111100111011101002?010000000101011101110120001000000011100010100100001021020222211010010011210100?01110?100???

Eugongylus_rufescens 10000?000000000?02?00120000000000??11010000?0?0400000001110??20?01301???0?01110?022???0102??0100000?01100002000013011000001000031000000002000000200000000110100111001020020?0000111012002022000000000011100011?21?0210101??100?000020110000?0??020?010003320??1?121?0000?01??0010?1001?0??2?20?0?0000012100000001000000000?110?00?0000?0??00010100000?0111001001010000?240?00010200300000?0010011011020010?000101000000001000?000043300000000000?000001101001000000300400000202?0?11000?????10000?10121000001110[01]102111100111011101002?010000000100011[12]01110100001000000001100010100100001021020222211010010011200100?01110?100??0

Tiliqua_scincoides 00010?000000000?00?00120000000000??11010000?0?0400000001110??2??0?301???0?011100022???0102??0010000?0110000200001401100100100003200000001?000000?00000001010100111001020020?0010111012002022000010000011100010?21?0210101??100?000020120000?0??220?010003320??1?101?1000?01??0110?1001?0??2020?0?0000012100000001000000000?110?00?0000?1??0001010100100011002001010000?20??10020200300000?0010021011010010?0001010000000000010000032200000000000?000001101001000000300410000202?0?11000?????10000?10121000011110010[12]11110011[12]011[01]01002?010000?0010001120111012000[01]0000000?1100010100120001020020222211010010011200100001100110?001

Shinisaurus_crocodilurus 100000200000000?00010??1100000000??10010010?0?0300000002120??2??0?00??0?0?000100012???0100??0200000?11100000000013001000000000011100000001000000200120001110100001001000000?000011101100102200000000001110001??11?0000101??101?000000101020?0??020?0100002?0?01?121?0000?00100010?2000?0001020?0?00000121000000010100000001110?00?0000?2??00010110010001010110010100100240?0001100000030000010010011020011000000?00000000001100000322000001100000000001100?011??0????0200000203?0?11001???2010000?10020000001011000000110010?000100000000000000011?0112011?0?000010000????1100011?00?00000?3101010100001001012000000000100111010?1

Xenosaurus_platyceps 100001200220000??0000101000000000??10020010?0?0100000002120??0000000000?0?010100022?0?0102??0000001?1110000000001301100000000001000000000100000120002000101010010011000?000?0000111011002022000000000011100010?21?0000101??101?000010100020?0??020?0100002?0?01?131?0000?01??0010?2000?0001020?0?00000121000000010000000001110?00?0000?0??00010100000000010000010100000240?0000000100030011010111011020011010000?0000100000010000033300000000000??0000010??011???????04000?0202?0?11030???2?1000??1????0??????10??0??????0???0?????0???0??00??00????11?011?0???????0??????11?001??00???0???310101110000?0?10120?????????0?1?10???1

Xenosaurus_grandis 100001200220000??0000101000000000??10020010?0?0200000002120??2??0?00??0?0?010100022???0102??0000001?1110000000001301100000000001000000000100000120002000101010010011000?000?0000111012002022000000000011100010?21?0000101??101?000010101020?0??020?0100002?0?01?131?0000?01??0010?2000?0001020?0?0100012100000001000000000?110?00?0000?0??00010100010?0001000001010000?240?00000001000300110101110110200110200001000010000000?000033300000000000?10000010??011??000000400000202?0?11030???2?10000?0[01]??0000001010000001110010100010000[01]001000000010001100111010000100000000110001010010000??3101010100001001012000?0000010011101001

Pseudopus_apodus 10001?000000000000000110000000000??00010000?0?0?40000002120??0000210100?0?011100012?0?0102??0100000?01100000000014011000000000010000000001000001200010000120000111001000010?0000111011001024000000000011100110?21?0001001??101?000000001020?0??020?0100002?0?000121?0000?00000010?2000?00?2020?0?00000121000000010000000001110?00?0000?0??0001011101000001001001010000?040?0111200100020011010110010020011000000?000110001001?00002220000001000000000011010011??000010420030213???10031???2??0000?31??0100001000?0000010000?3??????????0100[01]???1?????????????????????????????????????????10210111111010100101200000000010?11101??0

Peltosaurus_granulosus 100000200020000?01?00100000000000??10040000?0?0?10000002120??20?0?00??0?0?0011?0012???0102??0100000?011000?1000013011000000000010000000001000001200020?00010000111001000010?000011101100?02??000?0?00011?00010?2??0010100??101?000?00101020?0??020?0100002?0?000131?0000?00000010?2000?0001020?0?0100112?000000?10000000001?????0?0000?2???00101110?0000??0?000?0100000140?00100001000200100100100110200110200001000110001001000003330000001000000000???????1??????????????02010??11?????????0?????????0??????????000??1001?1????????????????????????????????????????????????????????????21210111111010???????????????????????????

Helodermoides_tuberculatus 1000??2?0000100?00000101000000000??00040000?0?0?40000002120??20?0200000?0?0011?00?2???0102??010?000?01100002?00013011000000000011000000001000001200121000010000101001001000?00101??0?100?0?????????00011100010?2??0010101???01?000?0???1020?0??020?010?002?0?0001?1?00?0?000?0010?2000?0001020?0?0??0?12?0000?0?????0????0?????????000???????????10???00????0??1???????140?001000010002?000??001201102?01??2??0010001100?10???0000?3?000000??000?0000?????????????????????????????1???????20?????????????????????????????????????????????????????????????????????????????????????????????212??1?1111010???????????????????????????

Anniella_pulchra 100000100000?00000?00000000100000??0124000000?0??0000001120??0000??0?00?0?1???????2?0??100??1?02000?0111?0??2?0013011000?00000012000000?01000001?00020?100??001?????100?010?0000111111001022001111000011100010?21?0010111??001?000000101020?0??020?0100023?0?01?121?0000?01??0011?22?0?11??020?0?11???12100000001020??1200?0???00?01?0?1??0010010?000?01010021110100010030?011110010000001001012001002001100000010000100010110000112110000110000?000001100?001???????0430030213???10001???2??001??????0[12]??????????1?????1????1?????1???01001???1???????????????????1?????????????????????102100?01110103010012000000?0010?11101??1

Celestus_enneagrammus 100010110020100000?00100000000000??01040000?0?0?20000002120??0000000000?0?001100012?0?0102??0010000?01100001000013011000000000010000000001000001200020000110000111001010010?0000111011001023000000000011100010?21?0010101??101?000010100020?0??020?010001200?01?111?0000?00000010?2000?0001020?0?00000121000000010000000001110?00?0000?0??00010110010?00010000010100000140?001120010003001001021001002001102000010001100010010000032200000010000?1000011010011??000110410000202???10010???2?10001110020000011[01]1001000111001110111010000010000000101011[01]0111002000100000000110001[01]10002000102101111110101000012???????0??????100??1

Elgaria_multicarinata 100000100000000?00000100000000000??10040000?0?0?10000002120??0000200000?0?000100012?0?0102??0100000?01100000000013011000000000030000000001000001200020000110000111001000010?0000111011001023000000000011100010?21?0010101??101?000010110020?0??020?010001200?01?111?0000?00000010?2000?0001020?0?00000121000000010000000001110?00?0000?0??00010110010?0001000001010000?140?01102001000300100102120100200110200101000110001001?000033300000000000?1000011010011??00001040000000200?10001???2010000?10020000001110010001110011101110100010000000001010111011100000010000000011000101000000010210111111010100101200000000010011101000

Gobiderma_pulchrum 1000001?000000??000001?0000010000??00120000?0?0400000002120??0000100000?0?0001?00?2?0?0102??000?001?01100000?00013011000000100011000000001000001200?2000012?000101001012000?00001?101100?0?????????000111001???2??00?1??1??10??000????01020????0?0?0100002?0?000131??000?000?0011?2000?000102??0?0??0?12??0000?????00000????????0?001??????00101?00?1?0???0??0??0100110040?0000?001010110?1010??20100200111?0000000000000001100001322100111??01?00000???????????????????????????0?1?0????????0?????????0????????????????????????????????1??0???????????????????????????????????????????????2001?111?0?0???????????????????????????

Estesia_mongoliensis 100000100000000?020101?1?0001???0??00120000?0?04000??002120??2??0?000?0?0?00?1?00?2???0102??010?000?0111?000?0001301?1000001000120?000??01000001??0?2100012??00101001010000?00001?10110??0???????0?0001????????????01101????01????00?10???0????020??100002?0?0001?1?0000?00000011?2000?10010???0?????????000000?1000?010001?????0?0010?????00??1000?0000010?30?10100110040?0000?0?10?0?????01???????020?111?0000??00??????011002023221001111?01000000????????????????????????0????100??????????????????????????????????????????????????????????????????????????????????????????????????????1??0?00????????????????????????????????

Aiolosaurus_oriens 110?10100000000?02?101?1000?1?000?????????0?0????0???????????0000100000???0001?00?????0???????????????1???0???00??0111000001000??00??0000100001??00??0?0?1????0?0100101?00??0000110011?0?0?????????0??11?0???0?2???0?1??1??1???0?0?????1?????????0??10???????????????????????0??1?2??0???????????0??0?1???????????????????????????????????????????????????????????00110040?0000?0010100?0?1010112010120011110000000000000001100202222100111??010?0000?????????????????????????????????????????????????????????????????????????????????????????????????????????????????????????????????????????????????????????????????????????????

Heloderma_horridum 1000000000001000020101?0000000000??0024000000?0??0000002110??0000101???????????????????102??0000000?0111?00000001?0110000001000120000000010000112000200001210000?1??1002100?0000111011001022000000000011100110021?0011001??101?000000101020?0??130?0100003?1?01?131?0000?01??0011?2000?1101020?0?0100112100000001010000000?110?00?0010?2??00010110010?00010030010100110041?0000?001010110?10100120100200111000000000000000010002023211001110001??0100011010011??000000410001212?1?10030???2010000?11??0100001?001100011001??2000100000000000000010?1110011?0?000010000000?1100010?00?00000?200101110000100101300010000010011101010

Heloderma_suspectum 10000?000000100?020101?0000000000??0024000000?0??0000002110??0000101???????????????????102??0000000?0111?00000001?0110000001000120000000010000112000200001110000????1002100?000011101100?023000000000011100110021?0011001??101?000000101020?0??130?0100003?1?01?131?0000?01??0011?2000?1101020?0?010011210000000101000000010???00?0010?2??00010110010?00010030010100110041?0000?001010110?1010012010020011100000?00000000001000202321100111?001??010001101[01]011??000000410001212?0?10030???2010000?11??01000011001100011001??2000000000[01]000000?0010?0111011100000010000000?1100011?00000000?2001011100002001013000?0000010011101010

Lanthanotus_borneensis 110000200000000?111101?1000010000??00130000?0?0??0000001120??0000101?0?????????????????100??0103000?0111?00000001?01110000?10003100000000101002120012100012?0000?1001000000?0000111011002022000000000011100211031?0010001??101?0000001010?0?0??020?0100003?1?001131?0000?00100011?2000?1101020?0?0000112100000001010?0001010???00?0011?2??00010110110000010130010100110041?0000?001010010?1010112010020111100000?000000000011?02022221001111001?00000011010011??01001041000120311010030???3120000?21??000000111011000110001?1000000000101000000010?0112011100200000000?00?1100011100020000?0101011100003?010140?0??00?1?0111101100

Saniwa 110010100000000?111101??000?10000??001200?0?0?04000000021?0??20001000?0?0?0001?00?????01?0??0?0?000?01100000?0001301010000010???1000000001010021200120000?2??00101?010?00???00001?10??0??0?????????000111?0110?0??0001011??101?000?00101020?0??0?0?0100001?1?000131?0000?000?0?11??000?1?0102????0?00?1210000?????1??0??????????0?0??0?????00????0??0?0???0?30?101?011?041?0000?001010110?10?011201?020?11???000000???????01100001332100111??01??00000??????1????????0400001202?101?030???3120000??00?000000????1?0??????????0?????0???00000??001010?12011?0?000010000????11?0011?00?0000?01100?0000?0????????????????????????????

Varanus_salvator 1100101001100000121101?1100010000??0?22000000?0?10000002120??2??0?00??0?0?000100?2????0100??0200000?0110000000001?011100000100011000000001020021200020010?2?000101001000000?0000111011003022000000000011100010?01?0011011??103?000000101020?0??020?0100001?1?01?131?0000?01??0011?2000?1101020?0?00000121000000010000000100110?00?0010?0??00010110110000010130010100110041?0000?001010110?1010112010020011100000?000000000011002023221001111001??000002100?111??11041040000221411010030???3120000?21??0000001011110000?0000?2000010011000000000010?011201110?000010000?00?1100010?00?00000?1101011110001?00024???????01?04?110???0

Varanus_acanthurus 111000200210000??21101?1000010000??0?22000000?0300000001120??2??0?00??0?0?000100?2????0100??0000000?0110000000001?011100000100010000000001020021000020010?2?000101001001000?0000111011004022000000000011100010?31?0011011??103?000000101020?0??020?0100001?1?01?131?0000?01??0011?2000?1101020?0?0000012100000001010?000100110?00?0011?0??00010110110000010130010100110041?0000?001010110?1010112010020011100000?000000000011?02022221001111001??000002100?101??11041040000220311010030???3120000?21??000000111111000100000?2000100011000000000010101120111000000100000000110001010000000??0100?0000?001001024???????01?04?110???0

Varanus_exanthematicus 1100002001100000?21101?1100010000??0?22000000?0400000002120??20?0?00??0?0?000100?2????0101??0000000?0110000000001?010100000100010000000001020021200120010?2?000101001000000?0000111011002022000000000011100110?01?0011011??103?000000101020?0??020?0100001?1?01?131?0000?01??0011?2000?1101020?0?00000121000000010000000101110?00?0010?0??00010100110000010130010100110041?0000?00101021001010112010020011100000?000000000011000023220001101001??000002100?111??110?0040000221411010030???3120000?2001000000101111000100000?2000010011000000000010?011201110?000010000?00?1100011100?4000??1100?0100?001001024??????001?04?1101100

Anelytropsis_papillosus 100000120010000002?00121000100000??01040000?0?0??00001011?0??0000?01?0?????????????????100??0?10000?11?1?0002?0014011000?00100010000000?1?0001????????????????????????????????????1112002023011112000011100010?21?0010121??100?00000010?0?0?0??2?0?0000033?0??1?111?1000?01??0111??2???00?2?20?0?0101112000000000?2???220??0??1?0?01?0?2??0010013??1100?110122?0010100?240?0?01020?3?1?????1???2101?0[012]1?10?0001010000000000011000121210000110000?0000000???001??0????0441020210???1?010??????00???????????????????1?????1??????????????????????1????????11??????0??????????????????????????0000?0000?00???1000123011??01000?10????

Dibamus_novaeguineae 100000220010100002?00121200100000??01040000?0?1??00001011?0??1?????1???????????????????100??0?11000?11?1?1?02?0014011000?00100000000000?1?0001????????????????1???????????????????1112002024011112000011100010?21?0010122??200?00000010?0?0?0??2?0?000003320??1?111?1000?01??0111??2???00?2?21?0?1?????2000000000?2???2200?0??1?0?01?0?2??0010013??11?01110122?0010000?240?0?01020?3?1?????1???2101?021?10?0001011000000000011000132110000110000?000001100?101???????0441020210???1?010??????0000?31??41?0100?00??1?????1????0?????01??01000??01???????????????????0101112011?110??????????0000?0000?00??01000023??10?01000?100??0

Spathorhynchus_fossorium 110000200200000??0?00030000000000??0124100000????0000003??0??0000101?0?????????????????1?0??1?00000?0111?0??2?001401??00?0?10002200000??1?00?011?0002000012?101?1????0000?0???00?1111200?0240011?2?00011100010?2??0010101??110?000000100000?0???20?000001300??1?100?1001?01??02?1?22???0001020?0?1?????2??3000??102???22??12?0022?0100?0???2???13?02100????132310100000230?100101103000000001001001102011201001010000100010112000212100000110000?0000?????????????????????30?11???100????????0?????????????????????????????????????????????????????????????????????????????????????????????2000?0000?????1????????????????????????

Dyticonastis_rensbergeri 1100012?0100000?00?00030100000000??0124100000????0000003??0??0000101?0?????????????????1?0??1?00000?0111?0??2?001401?000?0?10002200000?0??000011?0002000012?101?1?0??0??0?0???00?1???????????????2?00011100010?2??0010111??110?000000100000?0???20?000001300??1?100?10?1?01??02?1?22???0001020?0?1??????0030000?102???2200?2?0022?0100?1???010013??21001??0?32310100000230?1001021030000001010111011020112010000100001000?01120002121000001?0000?0000?????????????????????????????1????????????????????????????????????????????????????????????????????????????????????????????????????????2000?0?000????1????????????????????????

Rhineura_floridana 1100012000?0000?00?0003?100000000??0124100000????0000003??0??1?????1???????????????????1?0??1?00001?01?1?0??2?001401??00?0?10002200000??1?000021?0002?01??20101?11???0000?0?000??11112000024001112000011100011?01?0010101??110?000000100000?0??220?000001300??1?110?1001?01??0211?22???0001021?0?1?????210300000102???210012?0022?01?0?0??0010013?021?0101013231010000?030?0?0101103??0???1????100110201110?001010000100000?120002111000001?0000?000001100?011???????0441030?11???10020???20?001??????02??????????1?????1??????????????0100????1???????????????????1???????????????????????2000?0000?00??110041200110?01000?101??0

Bipes_biporus 1001002002000000?1?00001000?00000??1134100000????000001???0??1?????1???????????????????1?0??1?00001?12?1?0??2?001301?000?0000012200000??1?0001????????????????1?0????0000?0?000??11112000024001112000011100010?21?0110011??11??00000010?000?0??220?000001300??1?121?1001?01??0211?41???01?2?20?0?1?????210310000102???210??2?1022?01?0?1??0010013??21?0?01013231010100?230?0?0201100?1?????1???10010010?10?2001110000000000012000131100000010000?000001100?01000111000441030?12???10020???3??0001030000110000?00110000201????1?????012?01001??000100002011100102001011?????????????????????0000?0000?00??100041200?100010?011010?0

Bipes_canaliculatus 100100200200000??1?00001000?00000??1134100000????000001???0??1?????1???????????????????1?0??1?00001?12?1?0??2?001301?000?0000012200000??1?0001????????????????1?0????0000?0?000??11112000024001112000011100010?21?0110011??110?00000010?000?0??220?00000130???1?121?1001?01??0211?41???01?2?20?0?1?????210310000102???210??2?1022?01?0?1??0010013??21?0?01013231010100?230?0?0201100?1?????1???10010010?10?2001110000000000012000131100000010000?000001100?01000111000440030?12???10020??????0000?30000110000?00110000101????1?????012?01001??000100002011100112001011?????????????????????0000?0000?00??110????????????????10???0

Trogonophis_wiegmanni 110100200200000??1?00001000000000??0124100000?????000004??0??1?????1???????????????????1?0??1?00000?02?1?0??2?001401?000?00001?2?00000??1?0001????????????????1?0????02???0???0??11112002024001112000011100010?11?0010011??110?00000011?000?0??220?0000013?0??1?111?1001?01??0211??001?01?2?20?0?1????1200300000102???210012?1022?01?0?0??0010013?021[01]0101013231010100?030?0?0203000011????1?????000011100??0?1110?1??????00120001211011000??1???000001100?0101011?0004300?0?12???1?030??????001??????02??????????1?????1????1?????1???0100????1???????????????????1???????????????????????0000?0000?00??11004???????0?????1101??1

Diplometopon_zarudnyi 110100200200000??1?00001000000000??0124100?00?????00?004??0??1?????1???????????????????1?0??1?00000?12?1?0??2?001401?000?00002??????????1?00?1????????????????1??????1????????????1112000024001112000011100010?21?0010111??010?00000011?000?0??220?0000013?0??1?111?1001?01??0211??001??1?2?20?0?1?????210310000102???2100?2?1022?01?0?0??0010013?021?0101013231010000?030?0?0203000011????1???10000011200??02?11??1??????00120001111011000??1??0201001100?010101????0440030?11???10030??????0000?31??01?0100?????1?????1????1?????1???0100????1???????????????????1???????????????????????0000?0000?00??110????????????????10???0

Geocalamus_acutus 110000200200?00??1?010?1000000000??0124100000?????000004??0??1?????1???????????????????1?0??1?00000?02?1?0??2?0014?0?000?00001???0000???1?0001????????????????1??????1????????????0112000024001112000011100010?21?0010011??112?00000?10?020?0??220?0000013?0??1?131?1001?01??0211?41???01?2?20?0?1?????210300000102???210??2?1022?01?0?1??02???13?021?0?01013231010000?220?0?0203000?1?????00000?000010?10?2021110?1??????001200011110000?110000?000001000?0000011100??????0??????1?0?0??????001??????0101?00?00??1?????1??????????????0100????1???????????????????1???????????????????????0000?0000?00??110????????????????10???0

Amphisbaena_fuliginosa 100000200100000?01001001100100000??0024100000????0000004??0??1?????1???????????????????1?0??1?00000?02?1?0??2?001401?000?0010011200000??1?0001???00???????????1??????1????????????011200?024001112000011100011?21?0010111??112?000000100000?0??220?0000013?0??1?111?1001?01??0211?42???01?2?20?0?1?????200300000102???210??2?1022?01?0?1??02???13?021?0?01013231010100?240?0?0203000?1?????000010001010?10?202?110?1??????0012000221100000010000?00000110??01000????0?4200?0?12???100?0???3??00???????????????????1?????1??????????????????????1????????????????????????????????????????????000?0000?00??100041200110?01000???1??0

Najash_rionegrina ?????????????????????????????????????????0????????????0????????????????????????????????1?0001?0100000111????20????????????????????????????????????????????????1??????0?200000?00???11300?0?????112????????????????????????????????????????????????????????????????????0????????????????????????????????0?011000??????01?0??0????1?0??000???1???11??0??0??????1?10101????200???????00?0??????????????????????????????????????????????2?10????????????????????????????????????????1013030???20?0?1??????????????????1?????1????0?11??01??01001???1???????????????????010?????????????????????0??????00??????????????????????????????

Dinilysia_patagonica ????????10??????02011????001?0?10??00120000???1??01100022?000000??0000300010???00??40??100001?0100000111?00?2?00?11??11300000002000010201?0011????????????????1??????002000?00?0?1111300?0??0?1112?0??1????????????01??????????????0?10???0?0?0020?01000?3?0?0011?1000000?0100?100200000?0101?1001?????0?011010??????01?00?0??011?01000000?1???10?000000??0?210111???1102??0000?000010?13?101101202?020?10??00?01001??????0???2?00?2?110011?????0000?????????????????0??????2??01?130?????20?0?????????????????????????????????????????????????????????????????????????????????????????????0?00?0000??????????????????????????????

Leptotyphlops_dulcis 100001211??0100111011????001001?0??00241000?0?2??00?10012?0101?????1???????????????????100000???0?002??1?1??2?000?1??012000000?2000010201?00?1????????????????1??????1????????????01130040?4001012101?1100?2100210100000200201?0?000010?0001010??0?0100103?0??1?13100?000?1??1???????????????????1?????0010001000????1???010??011?01?0000012???11??01001?2012?01111??1101??0??0?000010?13??01101201001?210??0?0010000000000??????00011000011?01??00?002100??01???????0444040311?1013030000???001??????02??????????1?????1????0?1???01??01001??01???????????????????011?????????????????????0000?0000?00??11024???0??001?04?1101??0

Typhlops_jamaicensis 1000?011???0100101011????000000?0??00241000?0?2??100?0012?0?01?????1???????????????????100000???0?0011?1?1??2?0?0?????1?????00?20000?02?1??0?1????????????????1??????1????????????111300402400111210311100?2100110100?002002?1?0?0??0?0????101??4????0110?????1?1310??000?1??1?????????0?????????1?????001000?000????1???010??011?01?0010012???11??0000102013?11111??11040?0??1?0002?1??0??01000?010010110??000011000000000?????0?0101000011001??00?0020?0??01???????04441?0311?1013030000???001??????02??????????1?????1????1?????0???1???????1???????????????????1???????????????????????0000?0000?00??110????????????????1?1??0

Liotyphlops_albirostris 100??121???0?10011?11????001001?0??00241000?0?2??01?10012?0101?????00?????10????0??4???100000????0002??1?1??2?0?0?1???3??0??00??20????3?1????1????????????????1??????1????????????11130000?40?10121021110002101310100?002002?1?0?00?010?0???01???0???0110?????1?131??0000?1??0010?021000??2?20?011?????10010?1100????1???0?0??011?01?001??12????1??00001??012?01111??1102??0??0?0000?1?????01????010000110??0?0010000000000?????0?0111000011000??00?0????????????????0444130311?1013030000???0????????0??????????????????????0?????1???0???????1???????????????????1???????????????????????0000?0000?00??100??????????????????????

Typhlophis_squamosus 100???21???0?10011?11????001001?0??00241000?0?2??01?10012?0101?????00?????10????0??4???100000???0?002??1?0??2?0?0?1???3??0??00??20????3?1????1????????????????1??????1????????????11130000?40?10121021110002100310100?002002???0?00??10?0???01???0???0110?????1?131??0000?1??0010?021000??2?20?011?????10???0?200????1???0?0??011?01?001??12????0??00001??012?01111??1102??0??0?0000?1?????01????010000110??0?0010000000000?????0?0111000011000??00?0????????????????0444130311??01?0??000???0????????0????????????????????????????????????????????????????????????????????????????????????0000?0000?00??100??????????????????????

Anomochilus_leonardi 11000?0110110000010111?100010001000002311000121??01010013?0001?????1???????????????????100000?01000011?1?2??2?000?1??020000100?2000010201?0011????????????????1??????1??????0?????11130000220?1112100011000211111010100021120101?000010?0000010041?0100003?00?1?131000000?1??001???00000??2?20?0?1?????0001101000????11?0000??011111?0010012???11??0100002012121111111101?000?0?000010?13??01101200010?200?210011?000000000???02000111000111?00??0000????????????????0????30311???1?0?0000???0????????0??????????????????????0?????0???01??????1???????????????????1???????????????????????0000?0000?00??110?????????????????????0

Anilius_scytale 1100??0110110000010111?100010001000002311000301??01010023?0001?????1???????????????????100001?01000001?1?2??2?000?1??123000000?2000010201?0021????????????????1??????000000?0000?111130000210011121001110002111310101?0020020101?000010?030001004200100003?00?01131000000?01000100100?10??2?200001?????0001101000????11?0010??011111?1100012???11?00100002012121111011101?00?02?1000?1?????1???0?000100?00?211011?01??????010?22002221000111101?1000001??0??01???????0444230311?1013030000???001??????02??????????1?????1??????????0???01??????1???????????????????0???????????????????????0000?0000?00??11024????????1?0???101??1

Cylindrophis_ruffus 1100??011011000001011????0010001000002311000121??010100?3?0001?????00?31?010????0??1?0?100001??1000001?1?20?2?000?1??123000000??000110201?0011????????????????1??????002010?0000??1113000020101?12100111000211131010100020120101?00?010?030001004200100003?00?01131000000?01000100011010??2?200001?????0001101000????11?0000??011011010?0012???11?00011002012121111011101?000?1?000010?11?1011012000000200?211011?01??????0???22000221000111101?000001???0??01???????0444030311?1013030000???001??????02??????????1?????1????0?1???0???0100????1???????????????????0???????????????????????0000?0000?00??11024???????01?04?110???1

Uropeltis_melanogaster 1000001100110000010111?110010001000002311000122??01010013?0001?????1???????????????????100001??200002??1?2??2?00??1??023000000??000011201?0011????????????????1??????1????????????111300002400111210001100021110101000?021120001?000?10?0??00100?1?0100033000?1?131000000?1??00100100000??2?200001?????2001?0?000????11?0??0??011011?0000012???12??0??0?02012121111111101?000?1?000010?11??011012000100200?010011?01??????0???10000211100111101??00001???0??01???????04430?0311?1013030000???001??????02??????????1?????1??????????????????????????????????????????????????????????????????0000?0000?00??110????????????????1?????

Xenopeltis_unicolor 1100??011011000002011????0010?0?1000?2310000221??01010013?0001?????10??????????????????100001?01000011?1?2??2?000?2????3000000??00?2102?1?0011????????????????1??????002030?010???11130120200011131001110012111011101?0020120?02?00?010?030?0100420010110???0?02131100001?02000101011010??2?201111?????0002201000????11?0010??01101211000012???10?000000020121011110?1101?0???0??00010??0??01000?1??2??????2110?1?0000?000000?200?4441100101101?1000001??0??01???????0444030311?10130300002??001??????02??????????1?????1????1?????1???1???????1???????????????????1???????????????????????0000?0000?00??11024???0??001?04?110???0

Loxocemus_bicolor 1100??011011000002011????0010?010000?2311000211??01010013?0001?????00?31?110????0??3?1?100001??2000011?1?20?2?000?2???23000000??0000102?1?0011????????????????1??????002030?010???1113014020001113100111001211101110100020120?02?00?010?030?0100420010110??00?02131100001?02000101011010??2?200211?????0002201000????11?0010??01101211000011???10?00101002012101111111101?000?0?000010?11??01101?020100200?211011?00000000010?22001321100111101?100001???0??01???????04442?0311?101303000021?001??????02??????????1?????1????0?1???0???01??????1???????????????????0???????????????????????0010?0000?00??11024???????01?04?110???0

Xenophidion_acanthognathus 1100??001?11000002011????0010?0101?0?2?11000321??0?010012?0001?????1???????????????????100?01??200002??1?1??2?000?2???23000010??0000102?1?0011????????????????1??????0020301010???0113002020?00113100111001211121?100?0020120?02?00?010?03000100420010110???0?02131100101?02000101011010??2?200001?????0002201000????11?0000??01101100?00012???11?00?0110?012101111111101?0?0?0??00010?11?101100?02?2??????2100?1?0000?0000???23000321100111101?1000001?00??01???????0443030311?1013030000???001??????02??????????1?????1????1?????1???1???????1???????????????????1???????????????????????0000?0000?00??110??????????????????????

Casarea_dussumieri 1100??001011000002011????0010?0?2200?2310000311??01010012?0101?????00?31?010????0??4?0?100101??1000011?1?10?2?000?2???23000010??0000102?1?0011????????????????1??????0010300010???01130020201011131001110022111011101?0020120?02?00?010?0???0000420010110??00?01131100101?02000101011010??2?200101?????0002201000????11?0000??01101201000012???10?00001002012101111111101?000?0?000010?11?101101?1?010?100?211011?000000000???23000321100111101?10000????0??01?????????????0?????11????000????01??????02??????????1?????1????1?????1???1???????1???????????????????1???????????????????????0000?0000??0??110?????????????????????0

Haasiophis_terrasanctus ???00???10?????002111????001?0010??00??1??????????????022?0??1????000?3?0010???0?????0?1?0001?0?00000111?00???0???11?12300?00???00?0?0??1?0011????????????????1??????0020?0?0000??011300?0???????????1??????????????????????0????????10???????1??20???????????021311?0100?0200?101???0100?2?201??1?????0?011000????????????0??????1??100???1???1???0??0?????????0?1?111?1?0??00???0010?1??001?0??0101?0100??1???1?01??????0?0?2?00?331100111??1?100?0????????????????04420???13?1???130???21?101??????0???????????1?????1????0?11??01??01001???1???????????????????011??????1011??01???0???0000?0000?0????????????????????????????

Eupodophis_descouensis 11200???10??00?0????1??????????????0?????0??1?????????0????????????000?10010???0???4?1?????0??????????????0???0???1?????0??0000??????02??????1????????????????1??????00?????0100???1130??0?????????0?1????????????10?????00?0?????????0???0???1?420???11????????1?1????00?0??????1?1??0??????0?????????0???????????????????0??????1??10????????1????1?????????????1??????????????????0??????????????????????????1?????????0?????0003?1?0?11??????00?0????????????????04420??313?101313???????001??????0???????????1?????1??????????0???01001???1???????????????????011??????1?????????????????0?000000????????????????????????????

Pachyrhachis_problematicus 1??00???1?????????11????????????0??00?????????????????0?2?0??1????000?310010???00??4?1?1?0001???00000111?00???0???11?1?300?00???20?0?02?1?0011????????????????1??????0020?1?0100???1130?40?00?0???????????????????????????????????????0???????1??20?????????????131??0200????0?10101?0000?2?20???1?????0?0??0?0????????????0???????????????????1??????0???????????11?11?20??????0?00?0??????1?0???1??2???????1??1??1??????0??????0?22???01???????0??0???????????????????????313?1?1?1????????101??????0???????????1?????1????0?11??01??01001???1???????????????????011??????101?1??????????0000?0000??????????????????????????????

Exiliboa_placata 1000?0111?13001001011????0010?0?11?0?2210000302??01010012?0101?????00?31?010????0??1?0?100011??1000011?1?1??2?000?2???23000000??0000102?1?0011????????????????1??????002030?010???01130120201001131001110012111010100?0020120?02?00?010?03000110420010110???1?02131100101?02000101001010??2?200001?????0002201000????11?0000??01111211100012???10?00000102012101111111101?2?0?0??00010?11?101101?02?2??????211011?01??????0???20000331100111101?10000[01]??00??01???????0??????311???1???0000???001??????02??????????1?????1??????????????????????1???????????????????0???????????????????????0000?0000?00??110????????????????1????1

Ungaliophis_continentalis 1000??011?13001002011????0010?0101?0?2310000321??01010012?0101?????00?31?010????0??2?0?100001??1000011?1?10?2?000?2???21000000??0001102?1?0011????????????????1??????002030?010???01130140201001131001110012101010100?0020120?02?00??10?03000110420010110???0?02131100101?02000101?01010??2?201001?????0002201000????11?0010??01111211100012???11?0000010?012101111111101?1?0?0??00010??1??01100?02?2??????211011?01??????0???20000221100111101?10000????0??01???????0??????311???1???0000???001??????02??????????1?????1????0?????0???0???????1???????????????????0???????????????????????0000?0000?00??110????????????????1????1

Eryx_colubrinus 1100??01??12?1?002011????0010?0?1000?2311000303??01?10012?0101?????00?31?010????0??4?0?100001??1000111?1?10?2?000?2???23000000??000??02?1?0011????????????????1??????002030?010???01130140201001131001110012110010100?002012??02?00?010?030?0210420?????????0?02131100201?02000101000000??2?201001?????000?202000????11?0000??01111201100111???10?00000002012101111011101?100?0?000010?11?101101?000100100?211011?01??????0???23000221100111101?100001???0??01???????0444030311?101303011121?001??????02??????????1?????1????0?1???0???0???????1???????????????????0???????????????????????0000?0000?00??11024???????01?04?11?1??1

Calabaria_reinhardtii 1100?0211??201?102011????0010?010000?2311000302??01010012?0001?????00?3??010????0??4?0?100?10??1000011?1?00?2?000?2???23000000??0000102?1?0011????????????????1??????002030?010???01130110200001131001110002111010100?002012??02?00?010?0300011041?010110???0?1?131100101?1??00101000000??2?201001?????0002201000????11?0010??01111211100111???10?00101002012101111111101?100?0?000010?13?1011012000100200?011011?01??????0???23000321100111101??00001???0??01???????04442?0311?101303001020?001??????02??????????1?????1????0?1???0???0???????1???????????????????0???????????????????????0010?0000?00??11024???????01?04?11?1??0

Lichanura_trivirgata 1100??011??2001102011????0010?0?2200?2311000322??01010012?0101?????00?31?010????0??1?0?100011??1000011?1?10?2?000?2???23000000??0000102?1?0011????????????????1??????002030?010???01130130200001131001110012110010100?002012??02?00?010?03000110421??0110???0?02131100101?02000101000000??2?200001?????0002?00000????11?0000??01111211100012???10?00000002012101011011101?1?0?0??00010?11?101101?00?2??????111011?01??????0???23000321100111101?100001???0??01???????0444230311?101303011121?001??????02??????????1?????1????0?1???0???0???????1???????????????????0???????????????????????0000?0000?00??11024???????01?04?1101??1

Epicrates_striatus 1100??001?13001002011????0010?0?2200?2211000212??01010012?0001?????00?31?010????0??3?1?100001??1000111?1?00?2?000?2???23000000??0003102?1?0011????????????????1??????002030?010???01130140201001131001110012111011100?0020120?12?00?010?03000110421010110???1?02131100201?02000101011010??2?201001?????000?202000????11?0010??01111201100111???10?00101002012101111111101?100?0?000010?11?1011012000100100?211011?01??????0???23000321100111101?100001???0??01???????04442?0311?101303000021?001??????02??????????1?????1????0?1???0???0?0?0???1???????????????????011?????????????????????0000?0000?00??11024???????01?0??110???1

Boa_constrictor 1100??001?12001002011????001000?220002?11000213??01010012?0001?????00?31?010????0??4?1?100001??1000111?1?00?2?000?2???23000000??0003102?1?0011????????????????1??????002030?010???01130?20203001131001110012111011100?002012??02?00?010?0???001042101012????1?02131100201?02000101011010??2?201001?????000?202000????11?0010??01111201100111???10?00011002012101111101101?100?0?000010?11?1011012000100100?211011?01??????0???23000321100111101?100001???0??01???????04442?0311?101303000021?001??????02??????????1?????1????0?1???0???0???????1???????????????????0???????????????????????0000?0000?00??11024???????01?04?11?1??1

Aspidites_melanocephalus 1100??011?11000002011????0010?0111?0?2311000221??01010012?0001?????00?31?110????0??4?1?100001??2000011?1?00?2?000?2???23000000??0003102?1?0021????????????????1??????002031?010???01130140202001131001110012111011100?0020120?02?00?010?0???0010420010120???0?02131100201?02000101011010??2?200211?????000?202000????11?0010??01301200101011???11?0010?002012101111111101?100?0?000010?11??01100?000100100?211011?01??????0???23000221100111101?100001???0??01???????04442?0311?1013030000???001??????02??????????1?????1????0?????0???0???????1???????????????????0???????????????????????0010?0000?00??11024???????01?04?11????0

Python_molurus 1100??011?11000002011????0010?0111?0?2211000221??02010012?0001?????00?31?110????0??4?1?100001??2000111?1?0??2?000?2???23000000??0003102?1?0021????????????????1??????002030?010???01130140201001131001110012111010101?0020120?02?00?010?0???0010420010120???0?02131100201?02000101011010??2?201211?????000?202000????11?0010??01301201101011???10?00001002012101111111101?100?0?000010?11??01101?000100100?211011?01??????0???23001321100111101?100001???0??01???????04442?0311?101303000021?001??????02??????????1?????1????0?1???0???0???????1???????????????????0???????????????????????0010?0000?00??11024???????01?04?11????0

Trachyboa_boulengeri 1100??011?13001002011????0010?0?2200?2310000222??02010012?0001?????00?31?110????0??4?1?100111??200002??1?0??2?000?2???21100000??0000102?1?0011????????????????1??????0020300010???01130140202001131001110012111310100?0020020?12?00?010?031?0110420010100???0?02131100201?02000101011000??2?200201?????0002201000????11?0000??01301211100012???11?00001102012101111111101?2?0?0??00010?11?101100?02?2??????2130?1?000000000???13000231100111101?10000????0??01???????0441030311?1113030000???001??????02??????????1?????1????0?????0???0???????1???????????????????0???????????????????????0000?0000?00??110????????????????10???1

Tropidophis_haetianus 1100??011?13001002011????0010?0?2200?2310000222??02010011?0001?????00?31?110????0??4?1?100111??200002??1?0??2?000?2???21100000??0000102?1?0011????????????????1??????0010300010???01130140202001131001110012111310100?0020020?12?00?010?031?0110420010100???0?02131100201?02000101011000??2?200101?????0002201000????11?0000??01301211100012???11?00001002012101111111101?2?0?0??00010?11?101101?02?2??????2130?1?000000?00???13000331100111101?10000[01]???0??01???????04441?0311?111303000021?001??????02??????????1?????1????0?1???0???0???????1???????????????????0???????????????????????0000?0000?00??11024???????01?04?1101??1

Xenodermus_javanicus 1110??011??3001001111????0010?0?23?0?2?11000301??01010012?0101?????00?31?110????0??4?1?100101??200002??1?00?2?000?2???23100000??0010102?1?0021????????????????1??????0020311010???01130140202001131001110022111011101?0021130?02?0[01]?010?0???001042001???????0?02131200201?02100101021010??2?201211?????0002201000????11?0000??0110110000?012???10??0101102012101111111101?2?0?0??00010?1??101101?1??2??????113011?000000000???20000331100111101?0000001000??01???????0443030312?111303000021?001??????02??????????1?????1????1?????1???1???????1???????????????????1???????????????????????0000?0000?00??11024??????001?04?11?1??0

Acrochordus_granulatus 1000??021????1?002111????000????23?0?2?11000322??01?100?1?0101?????00?31?110????0??1?1?100?10??200002??1?00?2?000?2???23100000??000??12?1?0021????????????????1??????0020311010???01130?40203001131001110022110011100?0031130?02?00?010?0???000042001?10????0?02131200201?02000101021000??2?200201?????0002201000????11?0?00??03311210000012???10??0000??20?2111111101101?1?0?0??00010?11?101100?1??2??????2130?1?01??????0???23000321100111101?1000001??0??01???????0444000314?111303000021?001??????02??????????1?????1????1?????1???1???????1???????????????????1???????????????????????0000?0000?00??11024???????01?04?11?1??1

Pareas_hamptoni 1100??001??4000001111????0000?0?13?0?2311000302??01010011?0121?????00?31?010????0??2?0?100100??200002??1?00?2?0?0?2????1100000??2010112?1?0021????????????????1??????001030?000???01130240203001131001110022110011101?0031130?22?00?010?0???0000?2101?110???0?02131200201?02000101021010??2?201211?????0002201000????11?0000??01101210000012???10??000110201210111111110202?0?0??00010?1??10120??1??2??????2130?1?000000000???1??00221100111101?10000[01]???0??01???????0444040311?1013030000???001??????02??????????1?????1????1?????1???1???????1???????????????????1???????????????????????0000?0000?00??110?????????????????????0

Lycophidion_capense 1100??001??4000001111????0000?0?23?0?2311000301??01010012?0021?????00?3??010????0??2?0?100101??100002??1?1??2?000?3???23100000??2010112?1?0021????????????????1??????0020310010???0113024020300113100111001210001?100?0031130??3?00??10?0???0000420010110???0?02131200201?02000101021000??2?200111?????0002201000????11?0010??01101210000012???10??0001102012101111111101?0?0?0??00310?1???0?20??1??2??????2130?1?000000000???23000331100111101?0000001??0??01???????04441?0311?1?13030000???001??????02??????????1?????1????1?????1???1???????1???????????????????1???????????????????????0000?0000?00??11024???????01?0????????0

Aparallactus_werneri 10000?0010??000001011????0000?0?0000?2211000301??01010013?01?1?????00?31?010????0??0?0?100100??10000???1?10?2?000?3??023100000??0010112?1?0021????????????????1??????02003??0?0???01130?10210011131001110022110011101?0031130??3?00?010?0300010042001?11????0?02131200001?02000101021000??2?200111?????2002201000????11?0??0??01?011?0001012???11?00001?02012101111101101?2?0?0??00210?13?101201?1??2??????2130?1?000000000???24200231100111101?1010002000??01???????04420?0311?1013030000???001??????02??????????1?????1????1?????1???1???????1???????????????????1???????????????????????0000?0000?00??11024????????1?0???13???0

Atractaspis_irregularis 110??02010??010001111????0000?0?0000?2311000321??00010012?0101?????1???????????????????100100??200002??1?1??2?0?0?????4????000??0000112?1?0021????????????????1??????0010310010???01130?40?0401113100111002211001110??0031130??3?00??10?0300010????01?11??????021312?0201?1??0010?021000??2?200211?????0002101000????11?0010??01301200000012???11??0100102012101111111100???0?0??00210?11??01201?1??2??????0130?1?01??????0??????0010???01?110????200????0??01???????0444230311?1?13030000???001??????02??????????1?????1????1?????1???1???????1???????????????????1???????????????????????0000?0000?00??11024????????1?0???13???0

Causus_rhombeatus 110???001?14000002111????0000?0?2210?2?12001223??02010011?0121?????00?31?010????0??4?0?100100??100002??1?0??2?0?0?????4?????00??0000212?1?0021????????????????1??????0020310010???0113024020400113100111002211031110??003113??12?00??10?0???02?04?0?????????1?02131100201?0200011?021010??2?200211?????0002101000????11?0000??011?1210300012???10??0001102012101111111100???0?0??00210?????01200?1??2???????130?1?001000000???????0131100111101?0020001?00??01???????0442030311?1113030000???001??????02??????????1?????1????1?????1???1???????1???????????????????1???????????????????????0000?0000?00??11024????????1?0???13???0

Azemiops_feae 110??0101?14001002111????0000?0?1200?2311000322??01010012?0101?????00?31?110????0??4?1?100100??200002??1?00?2?0?0?????4?????00??1010212?1?0021????????????????1??????0020310010???0113024020200113100111002211001110??0031130?12?00?010?030?01?0421??0110???1?02131100201?0200011?021000??2?200211?????0002201000????11?0010??01[13]?1210000012???10??0001102012101111011101?2?0?0??00010??1?101200?1??2??????2130?1?001000000???????0121100111101?1020001000??01???????04430?0311?1113030000???001??????02??????????1?????1????1?????1???1???????1???????????????????1???????????????????????0000?0000?00??110????????????????1????0

Daboia_russelli 110??0101?14001002111????0000?0?23?0?2?12000323??02110011?0111?????00?31?010????0??2?0?100100??100002??1?0??2?0?0?????4?????00??0013112?1?0021????????????????1??????0020310010???0113024020400113100111002211001110??003113??22?00??10?0???02?04?1?????????1?02131100201?0200011?021000??2?200211?????0002201000????11?0010??011?1212300012???10??0102102012101111101101???0?0??00210??1??01200?1??2??????2130?1?001000000???????0121100111101?002001??00??01???????0443030311?1113030000???001??????02??????????1?????1????1?????1???1???????1???????????????????1???????????????????????0000?0000?00??11024???????01?04?11????1

Agkistrodon_contortrix 100??0101?14001002111????0000?0?2210?2?110003?3??02110011?0111?????00?31?010????0??0?0?100100??100002??1?0??2?0?0?????4?????00??0010?12?1?0021????????????????1??????0020310010???0113024020300113100111002211001110??003113??12?0[01]??10?0???00?0421???12????1?02131100201?0200011?021000??2?200111?????0002201000????11?0010??011?1212300012???10?00002102012101111101101?2?0?0??00310??1??01200?1??2??????2130?1?001000000???????0121100111101?0020001?00??01???????0442030312?111303000021?001??????02??????????1?????1????1?????1???1???????1???????????????????1???????????????????????0000?0000?00??11024???????01?04?11????1

Bothrops_asper 110??0101?14001002111????0000?0?2210?2?10000302??02110011?0011?????00?31?010????0??0?0?100100??100002??1?00?2?0?0?????4?????00??0010?12?1?0021????????????????1??????0020310010???01130240?0400113100111002211001110??003113??12?00?010?0???00?0421???12????1?02131100201?0200011?021000??2?200211?????0002201000????11?0010??011?1212300012???10??0002102012101111001101???0?0??00310?????01200?1??2??????2130?1?001000000???????0121100111101?002001??00??01???????04441?0312?111303000021?001??????02??????????1?????1????1?????1???1???????1???????????????????1???????????????????????0000?0000?00??10024????????1?0???1????1

Lachesis_muta 110???001?14000002111????0000?0?23?0?2?10000302??02110011?0111?????00?31?010????0??0?0?100100??100002??1?0??2?0?0?????4?????00??0000?12?1?0021????????????????1??????0020310010???0113024020300113100111002211001110??003113??12?00??10?0???00?0421???12????1?02131100201?0200011?021000??2?200211?????0002201000????11?0010??011?1212300012???10??0002102012101111001101???0?0??00010?????01200?1??2??????2130?1?001000000???????0121100111101?0020002000??01???????0444230312?111303000021?001??????02??????????1?????1????1?????1???1???????1???????????????????1???????????????????????0000?0000?00??11024????????1?0???1????0

Naja_naja 1000??001?13001002111????0010?0?2200?2212001323??01010012?0121?????00?31?010????0??3?0?100100??100002??1?00?2?000?2???2?100?00??0012212?1?0021????????????????1??????0020310010???01130240203001131001110022110011100?0031130?12?00??10?030?0000?20010120???1?02131200201?02000101011?00??2?200211?????0002201000????11?0000??01101210000012???12?00001102012101111111101?2?0?0??00310?11??01201?1??2??????2110?1?000000000?????1?0121100111101?00200[01]??00??01???????04441?0312?111303000021?001??????02??????????1?????1????1?????1???1???????1???????????????????1???????????????????????0000?0000?00??10024???????01?04?113???0

Notechis_scutatus 110???001?14000002111????0000?0?2200?2211001323??01010012?0121?????00?31?010????0??2?0?100101??100002??1?00?2?000?2?????10??00??0010212?1?0021????????????????1??????0020310010???01130240203001131001110022110011101?003113??12?00?010?0???02?0420010??????1?02131200201?02000101011?10??2?200211?????0002201000????11?0000??011?1210000012???12?00101102012101111111101?2?0?0??00310?11??01200?1??2??????2110?1?00000?000?????1?0231100111101?002001??00??01???????04430?0311?111303000021?001??????02??????????1?????1????1?????1???1???????1???????????????????1???????????????????????0000?0000?00??11024????????1?0????????1

Laticauda_colubrina 1100??001??4000002111????0000?0?23?0?2?11000301??01110012?0021?????00?31?010????0??3?1?100001??100002??1?10?2?000?2???2?10??00??0003212?1?0021????????????????1??????0020310010???0113004020200???1001110020110011101?003113???4?00??10?0???020042001???????1?02131200201?02000101?00?00??2?201211?????000?201000????11?0000??01101100000012???10?00101102012101111101101?1?0?0??00310?13??01200?1??2??????0110?1?000000000?????1?0121100111101?1020001000??01???????0444230312?1113030000???001??????02??????????1?????1????1?????1???1???????1???????????????????1???????????????????????0000?0000?00??11024???????01?04?113???0

Micrurus_fulvius 1101??001?14000002111????0000?0?2200?2211000321??01010013?0021?????1???????????????????100001??100002??1?2??2?000?2???2?10??00??0013112?1?0021????????????????1??????0020310010???01130140202001131001110022110011101?0031130??4?00?010?030?0000?20010110???1?02131200201?02000101010?00??2?200111?????0002201000????11?0000??01311100001012???10?00101102012101111011101?2?0?0??00310?11?101200?1??2??????2110?1?000000000???????0121100111101?102000?000??01???????0444230312?1113030000???001??????02??????????1?????1????1?????1???1???????1???????????????????1???????????????????????0000?0000?00??11024???????01?0??11????0

Natrix_natrix 1100??001??4000002111????0100?0?23?0?2212001323??02010022?0121?????00?31?010????0??2?0?100101??200002??1?00?2?000?2???23100000??0010212?1?0021????????????????1??????0020311010???01130240204001131001110032110011101?0031130?22?00??10?0???0100420010110???0?02131200201?02100101022010??2?201211?????0002201000????11?0000??01301211201012???10?00001102012101111111101?2?0?0??00310?11?10120??1??2??????2130?1?000000000???24200331100111101?000000???0??01???????0443030312?1113030000???001??????02??????????1?????1????1?????1???1???????1???????????????????1???????????????????????0000?0000?00??10024????????1?0???13???0

Afronatrix_anoscopus 1100??001??4000002111????0100?0?23?0?2211001322??02010012?0121?????00?31?010????0??3?0?100101??200002??1?00?2?000?2???23100000??0010212?1?0021????????????????1??????0020311010???0113024020300113100111003211001110??0031130?22?01?010?0???010042001011????0?02131200201?02100101022010??2?200211?????0002201000????11?0000??01301211201012???10?00001102012101111101101?2?0?0??00310?1??101201?1??2??????2130?1?000000000???24200331100111101?000001???0??01???????04420?0311?1?13030000???001??????02??????????1?????1????1?????1???1???????1???????????????????1???????????????????????0000?0000?00??110????????????????1????0

Amphiesma_stolata 1100?0101??4000002111????0100?0?23?0?2212001323??02010012?0?21?????00?31?010????0??2?0?100100??200002??1?00?2?000?2???23100000??0010212?1?0021????????????????1??????0020310010???0113014020?001131001110032110011101?0031130?22?01?010?0???0100420010110???0?02131200201?02100101022010??2?201211?????0002201000????11?0010??01301211201012???10?00001102012101111111101?2?0?0??00310?1??101201?1??2??????2130?1?000000000???24200331100111101?001001???0??01???????0442030312?111303000021?001??????02??????????1?????1????1?????1???1???????1???????????????????1???????????????????????0000?0000?00??110???????????????113???0

Thamnophis_marcianus 1100??001??4000002111????0100?0?23?0?2212001323??02010012?0021?????00?31?010????0??2?0?100101??200002??1?00?2?000?2???23100000??0010212?1?0021????????????????1??????0020311010???01130140204001131001110032110011101?003113??22?00??10?0???010042001?110???0?02131200201?02100101022010??2?201211?????0002201000????11?0000??01301211201012???11?00101102012101111111101?2?0?0??00310?1??101201?1??2??????2130?1?000000000???24200331100111101?000001??00??01???????0442030312?111303000021?001??????02??????????1?????1????1?????1???1???????1???????????????????1???????????????????????0000?0000?00??11024???????01?04?113???1

Xenochrophis_piscator 1100??001??4000002111????0100?0?23?0?2212001323??02010012?0121?????00?31?010????0??0?0?100101??200002??1??0?2?000?2???23100000??0010212?1?0021????????????????1??????0020311010???01130240203001131001110032110011100?003113??22?00?010?0???0100420010110???0?02131200201?02100101022010??2?201211?????0002201000????11?0000??01301211201012???11?00101102012101111111101?2?0?0??00310?11??01201?1??2??????2130?1?000000000???24200331100111101?100001??00??01???????0442030312?111303000021?001??????02??????????1?????1????1?????1???1???????1???????????????????1???????????????????????0000?0000?00??110????????????????13???0

Lampropeltis_getula 1100??001?14000002111????0000?0?2200?2212001323??02010014?0121?????00?31?010????0??3?0?100101??200002??1?00?2?000?2???23100000??0010212?1?0021????????????????1??????0020311010???0113024020?001131001110032110011101?003113??22?00??10?0???0100420010110???0?02131200201?02100101021010??2?200211?????0002201000????11?0010??0130121100101[12]???10?00001102012101111111101?2?0?0??00310?1??10120??1??2??????2130?1?000000000???24000221100111101?100001??00??01???????04442?0312?101303000021?001??????02??????????1?????1????1?????1???1???????1???????????????????1???????????????????????0000?0000?00??11024???????01?04?11????0

Coluber_constrictor 1100??001??4000002111????0000?0?2200?2212001323??02010024?0121?????00?31?010????0??3?0?100101??200002??1?00?2?000?2???23100000??0011212?1?0021????????????????1??????0020311010???01130240203001131001110032110011101?003113??22?00?010?0???0000420010100???0?02131200201?02100101021010??2?200211?????0002201000????11?0010??01301210001012???10?00001102013101111111101?2?0?0??00310?1??101201?1??2??????2130?1?000000000???24000221100111101?1000002000??01???????04430?0312?101303000021?001??????02??????????1?????1????1?????1???1???????1???????????????????1???????????????????????0000?0000?00??11024???????01?04?113???0

Gueragama_sulamericana ??????????????????????????????????????????????????????????????????????????????????????????????????????????????????????????????????????????????????????????????????????????????????????????????????????????????????????????????????????????????????????????????????????????????????????????????????????????????????????????????????????????????????????????????????0100024??200202000???????0????????0?0??0??????????????????????????200000000000?1000?????????????????????????????????????????????????????????????????????????????????????????????????????????????????????????????????????????????????????????????????????????????

;

Ccode

-[/1 0 -[/1 1 +[/1 2 -[/1 3 -[/1 4

-[/1 5 +[/1 6 -[/1 7 -[/1 8 +[/1 9

-[/1 10 +[/1 11 -[/1 12 -[/1 13 -[/1 14

-[/1 15 -[/1 16 +[/1 17 -[/1 18 -[/1 19

-[/1 20 -[/1 21 -[/1 22 -[/1 23 +[/1 24

-[/1 25 -[/1 26 -[/1 27 -[/1 28 -[/1 29

-[/1 30 -[/1 31 +[/1 32 -[/1 33 -[/1 34

-[/1 35 -[/1 36 +[/1 37 +[/1 38 -[/1 39

+[/1 40 -[/1 41 +[/1 42 -[/1 43 +[/1 44

-[/1 45 -[/1 46 +[/1 47 +[/1 48 -[/1 49

+[/1 50 -[/1 51 -[/1 52 -[/1 53 -[/1 54

+[/1 55 -[/1 56 +[/1 57 -[/1 58 -[/1 59

-[/1 60 -[/1 61 +[/1 62 -[/1 63 +[/1 64

+[/1 65 +[/1 66 -[/1 67 -[/1 68 +[/1 69

-[/1 70 -[/1 71 -[/1 72 -[/1 73 -[/1 74

-[/1 75 -[/1 76 -[/1 77 -[/1 78 +[/1 79

-[/1 80 +[/1 81 +[/1 82 +[/1 83 -[/1 84

-[/1 85 -[/1 86 -[/1 87 -[/1 88 +[/1 89

-[/1 90 -[/1 91 +[/1 92 -[/1 93 -[/1 94

-[/1 95 +[/1 96 -[/1 97 +[/1 98 -[/1 99

+[/1 100 +[/1 101 -[/1 102 -[/1 103 +[/1 104

+[/1 105 -[/1 106 +[/1 107 -[/1 108 -[/1 109

+[/1 110 -[/1 111 -[/1 112 +[/1 113 -[/1 114

-[/1 115 -[/1 116 -[/1 117 -[/1 118 +[/1 119

-[/1 120 -[/1 121 -[/1 122 -[/1 123 -[/1 124

+[/1 125 -[/1 126 +[/1 127 +[/1 128 +[/1 129

-[/1 130 +[/1 131 +[/1 132 -[/1 133 -[/1 134

-[/1 135 -[/1 136 -[/1 137 -[/1 138 +[/1 139

+[/1 140 -[/1 141 +[/1 142 -[/1 143 -[/1 144

-[/1 145 -[/1 146 -[/1 147 +[/1 148 -[/1 149

-[/1 150 +[/1 151 -[/1 152 -[/1 153 +[/1 154

-[/1 155 -[/1 156 -[/1 157 -[/1 158 -[/1 159

-[/1 160 -[/1 161 -[/1 162 -[/1 163 -[/1 164

-[/1 165 +[/1 166 +[/1 167 -[/1 168 -[/1 169

-[/1 170 -[/1 171 -[/1 172 -[/1 173 -[/1 174

-[/1 175 -[/1 176 +[/1 177 -]/1 178 -[/1 179

-[/1 180 +[/1 181 -[/1 182 +[/1 183 +[/1 184

-[/1 185 +[/1 186 +[/1 187 +[/1 188 -[/1 189

-[/1 190 -[/1 191 -[/1 192 -[/1 193 -[/1 194

-[/1 195 -[/1 196 -[/1 197 -[/1 198 -[/1 199

-[/1 200 -[/1 201 +[/1 202 +[/1 203 -[/1 204

-[/1 205 -[/1 206 +[/1 207 -[/1 208 -[/1 209

-[/1 210 -[/1 211 -[/1 212 -[/1 213 -[/1 214

+[/1 215 +[/1 216 -[/1 217 -[/1 218 +[/1 219

-[/1 220 -[/1 221 +[/1 222 -[/1 223 -[/1 224

-[/1 225 -[/1 226 -[/1 227 -[/1 228 -[/1 229

+[/1 230 -[/1 231 -[/1 232 -[/1 233 -[/1 234

-[/1 235 -[/1 236 +[/1 237 -[/1 238 -[/1 239

-[/1 240 +[/1 241 -[/1 242 -[/1 243 -[/1 244

-[/1 245 -[/1 246 +[/1 247 -[/1 248 +[/1 249

+[/1 250 -[/1 251 -[/1 252 -[/1 253 -[/1 254

+[/1 255 -[/1 256 +[/1 257 -[/1 258 +[/1 259

-[/1 260 -[/1 261 +[/1 262 -[/1 263 -[/1 264

-[/1 265 -[/1 266 +[/1 267 -[/1 268 -[/1 269

+[/1 270 -[/1 271 -[/1 272 -[/1 273 -[/1 274

+[/1 275 +[/1 276 -[/1 277 -[/1 278 -[/1 279

-[/1 280 -[/1 281 +[/1 282 -[/1 283 +[/1 284

-[/1 285 -[/1 286 +[/1 287 -[/1 288 -[/1 289

-[/1 290 -[/1 291 -[/1 292 -[/1 293 -[/1 294

-[/1 295 -[/1 296 -[/1 297 -[/1 298 +[/1 299

+[/1 300 +[/1 301 +[/1 302 -[/1 303 -[/1 304

+[/1 305 -[/1 306 -[/1 307 +[/1 308 -[/1 309

+[/1 310 +[/1 311 -[/1 312 -[/1 313 -[/1 314

+[/1 315 -[/1 316 -[/1 317 -[/1 318 -[/1 319

-[/1 320 -[/1 321 -[/1 322 -[/1 323 -[/1 324

+[/1 325 -[/1 326 +[/1 327 -[/1 328 -[/1 329

-[/1 330 -[/1 331 -[/1 332 -[/1 333 -[/1 334

-[/1 335 +[/1 336 -[/1 337 -[/1 338 +[/1 339

-[/1 340 -[/1 341 +[/1 342 -[/1 343 -[/1 344

+[/1 345 +[/1 346 -[/1 347 +[/1 348 +[/1 349

-[/1 350 -[/1 351 -[/1 352 -[/1 353 -[/1 354

-[/1 355 -[/1 356 -[/1 357 -[/1 358 +[/1 359

+[/1 360 -[/1 361 -[/1 362 +[/1 363 -[/1 364

-[/1 365 -[/1 366 +[/1 367 +[/1 368 -[/1 369

-[/1 370 +[/1 371 -[/1 372 -[/1 373 +[/1 374

-[/1 375 -[/1 376 -[/1 377 -[/1 378 -[/1 379

-[/1 380 +[/1 381 -[/1 382 -[/1 383 -[/1 384

-[/1 385 -[/1 386 +[/1 387 +[/1 388 +[/1 389

-[/1 390 +[/1 391 -[/1 392 +[/1 393 -[/1 394

+[/1 395 -[/1 396 -[/1 397 -[/1 398 -[/1 399

-[/1 400 -[/1 401 -[/1 402 -[/1 403 -[/1 404

-[/1 405 -[/1 406 -[/1 407 -[/1 408 -[/1 409

-[/1 410 -[/1 411 -[/1 412 +[/1 413 +[/1 414

-[/1 415 -[/1 416 +[/1 417 +[/1 418 +[/1 419

+[/1 420 -[/1 421 -[/1 422 -[/1 423 -[/1 424

-[/1 425 -[/1 426 -[/1 427 -[/1 428 -[/1 429

-[/1 430 -[/1 431 -[/1 432 -[/1 433 +[/1 434

-[/1 435 -[/1 436 -[/1 437 -[/1 438 +[/1 439

-[/1 440 -[/1 441 -[/1 442 -[/1 443 -[/1 444

-[/1 445 -[/1 446 -[/1 447 -[/1 448 -[/1 449

-[/1 450 -[/1 451 -[/1 452 +[/1 453 +[/1 454

+[/1 455 +[/1 456 +[/1 457 +[/1 458 +[/1 459

-[/1 460 -[/1 461 +[/1 462 -[/1 463 -[/1 464

-[/1 465 -[/1 466 +[/1 467 -[/1 468 -[/1 469

-[/1 470 -[/1 471 -[/1 472 -[/1 473 +[/1 474

-[/1 475 +[/1 476 -[/1 477 -[/1 478 -[/1 479

-[/1 480 -[/1 481 +[/1 482 -[/1 483 -[/1 484

+[/1 485 +[/1 486 +[/1 487 -[/1 488 -[/1 489

-[/1 490 -[/1 491 -[/1 492 -[/1 493 -[/1 494

-[/1 495 -[/1 496 -[/1 497 -[/1 498 -[/1 499

-[/1 500 -[/1 501 -[/1 502 -[/1 503 -[/1 504

-[/1 505 -[/1 506 -[/1 507 -[/1 508 -[/1 509

-[/1 510 -[/1 511 -[/1 512 -[/1 513 -[/1 514

-[/1 515 -[/1 516 +[/1 517 -[/1 518 -[/1 519

-[/1 520 -[/1 521 -[/1 522 -[/1 523 -[/1 524

-[/1 525 -[/1 526 -[/1 527 +[/1 528 -[/1 529

-[/1 530 -[/1 531 -[/1 532 -[/1 533 +[/1 534

-[/1 535 -[/1 536 -[/1 537 -[/1 538 -[/1 539

-[/1 540 -[/1 541 -[/1 542 -[/1 543 -[/1 544

-[/1 545 -[/1 546 -[/1 547 -[/1 548 -[/1 549

-[/1 550 -[/1 551 -[/1 552 -[/1 553 -[/1 554

-[/1 555 -[/1 556 -[/1 557 -[/1 558 -[/1 559

-[/1 560 -[/1 561 -[/1 562 -[/1 563 -[/1 564

-[/1 565 -[/1 566 -[/1 567 -[/1 568 +[/1 569

-[/1 570 +[/1 571 -[/1 572 -[/1 573 -[/1 574

-[/1 575 -[/1 576 -[/1 577 -[/1 578 -[/1 579

-[/1 580 -[/1 581 -[/1 582 +[/1 583 -[/1 584

-[/1 585 -[/1 586 +[/1 587 +[/1 588 +[/1 589

-[/1 590 -[/1 591 +[/1 592 -[/1 593 -[/1 594

-[/1 595 -[/1 596 -[/1 597 -[/1 598 -[/1 599

-[/1 600 +[/1 601 -[/1 602 -[/1 603 -[/1 604

-[/1 605 -[/1 606 -[/1 607 -[/1 608 -[/1 609

;

Ancstates

-0 -1 -2 -3 -4 -5 -6 -7 -8 -9

-10 -11 -12 -13 -14 -15 -16 -17 -18 -19

-20 -21 -22 -23 -24 -25 -26 -27 -28 -29

-30 -31 -32 -33 -34 -35 -36 -37 -38 -39

-40 -41 -42 -43 -44 -45 -46 -47 -48 -49

-50 -51 -52 -53 -54 -55 -56 -57 -58 -59

-60 -61 -62 -63 -64 -65 -66 -67 -68 -69

-70 -71 -72 -73 -74 -75 -76 -77 -78 -79

-80 -81 -82 -83 -84 -85 -86 -87 -88 -89

-90 -91 -92 -93 -94 -95 -96 -97 -98 -99

-100 -101 -102 -103 -104 -105 -106 -107 -108 -109

-110 -111 -112 -113 -114 -115 -116 -117 -118 -119

-120 -121 -122 -123 -124 -125 -126 -127 -128 -129

-130 -131 -132 -133 -134 -135 -136 -137 -138 -139

-140 -141 -142 -143 -144 -145 -146 -147 -148 -149

-150 -151 -152 -153 -154 -155 -156 -157 -158 -159

-160 -161 -162 -163 -164 -165 -166 -167 -168 -169

-170 -171 -172 -173 -174 -175 -176 -177 -178 -179

-180 -181 -182 -183 -184 -185 -186 -187 -188 -189

-190 -191 -192 -193 -194 -195 -196 -197 -198 -199

-200 -201 -202 -203 -204 -205 -206 -207 -208 -209

-210 -211 -212 -213 -214 -215 -216 -217 -218 -219

-220 -221 -222 -223 -224 -225 -226 -227 -228 -229

-230 -231 -232 -233 -234 -235 -236 -237 -238 -239

-240 -241 -242 -243 -244 -245 -246 -247 -248 -249

-250 -251 -252 -253 -254 -255 -256 -257 -258 -259

-260 -261 -262 -263 -264 -265 -266 -267 -268 -269

-270 -271 -272 -273 -274 -275 -276 -277 -278 -279

-280 -281 -282 -283 -284 -285 -286 -287 -288 -289

-290 -291 -292 -293 -294 -295 -296 -297 -298 -299

-300 -301 -302 -303 -304 -305 -306 -307 -308 -309

-310 -311 -312 -313 -314 -315 -316 -317 -318 -319

-320 -321 -322 -323 -324 -325 -326 -327 -328 -329

-330 -331 -332 -333 -334 -335 -336 -337 -338 -339

-340 -341 -342 -343 -344 -345 -346 -347 -348 -349

-350 -351 -352 -353 -354 -355 -356 -357 -358 -359

-360 -361 -362 -363 -364 -365 -366 -367 -368 -369

-370 -371 -372 -373 -374 -375 -376 -377 -378 -379

-380 -381 -382 -383 -384 -385 -386 -387 -388 -389

-390 -391 -392 -393 -394 -395 -396 -397 -398 -399

-400 -401 -402 -403 -404 -405 -406 -407 -408 -409

-410 -411 -412 -413 -414 -415 -416 -417 -418 -419

-420 -421 -422 -423 -424 -425 -426 -427 -428 -429

-430 -431 -432 -433 -434 -435 -436 -437 -438 -439

-440 -441 -442 -443 -444 -445 -446 -447 -448 -449

-450 -451 -452 -453 -454 -455 -456 -457 -458 -459

-460 -461 -462 -463 -464 -465 -466 -467 -468 -469

-470 -471 -472 -473 -474 -475 -476 -477 -478 -479

-480 -481 -482 -483 -484 -485 -486 -487 -488 -489

-490 -491 -492 -493 -494 -495 -496 -497 -498 -499

-500 -501 -502 -503 -504 -505 -506 -507 -508 -509

-510 -511 -512 -513 -514 -515 -516 -517 -518 -519

-520 -521 -522 -523 -524 -525 -526 -527 -528 -529

-530 -531 -532 -533 -534 -535 -536 -537 -538 -539

-540 -541 -542 -543 -544 -545 -546 -547 -548 -549

-550 -551 -552 -553 -554 -555 -556 -557 -558 -559

-560 -561 -562 -563 -564 -565 -566 -567 -568 -569

-570 -571 -572 -573 -574 -575 -576 -577 -578 -579

-580 -581 -582 -583 -584 -585 -586 -587 -588 -589

-590 -591 -592 -593 -594 -595 -596 -597 -598 -599

-600 -601 -602 -603 -604 -605 -606 -607 -608 -609

;

xgroup

;

agroup

;

taxcode

+0 +1 +2 +3 +4 +5 +6 +7

+8 +9 +10 +11 +12 +13 +14 +15

+16 +17 +18 +19 +20 +21 +22 +23

+24 +25 +26 +27 +28 +29 +30 +31

+32 +33 +34 +35 +36 +37 +38 +39

+40 +41 +42 +43 +44 +45 +46 +47

+48 +49 +50 +51 +52 +53 +54 +55

+56 +57 +58 +59 +60 +61 +62 +63

+64 +65 +66 +67 +68 +69 +70 +71

+72 +73 +74 +75 +76 +77 +78 +79

+80 +81 +82 +83 +84 +85 +86 +87

+88 +89 +90 +91 +92 +93 +94 +95

+96 +97 +98 +99 +100 +101 +102 +103

+104 +105 +106 +107 +108 +109 +110 +111

+112 +113 +114 +115 +116 +117 +118 +119

+120 +121 +122 +123 +124 +125 +126 +127

+128 +129 +130 +131 +132 +133 +134 +135

+136 +137 +138 +139 +140 +141 +142 +143

+144 +145 +146 +147 +148 +149 +150 +151

+152 +153 +154 +155 +156 +157 +158 +159

+160 +161 +162 +163 +164 +165 +166 +167

+168 +169 +170 +171 +172 +173 +174 +175

+176 +177 +178 +179 +180 +181 +182 +183

+184 +185 +186 +187 +188 +189 +190 +191

+192

;

blocks 0;

proc/;
